# Flexible Coordination Network Exhibiting Water Vapor–Induced Reversible Switching between Closed and Open Phases

**DOI:** 10.1021/acsami.2c10002

**Published:** 2022-08-17

**Authors:** Mohana Shivanna, Andrey A. Bezrukov, Victoria Gascón-Pérez, Ken-ichi Otake, Suresh Sanda, Daniel J. O’Hearn, Qing-Yuan Yang, Susumu Kitagawa, Michael J. Zaworotko

**Affiliations:** † Department of Chemical Sciences, Bernal Institute, University of Limerick, Limerick V94 T9PX, Republic of Ireland; ‡ Institute for Integrated Cell-Material Sciences, Kyoto University Institute for Advanced Study, Kyoto University Yoshida Ushinomiya-cho, Sakyo-ku, Kyoto 606-8501, Japan

**Keywords:** sorbents, structural flexibility, water sorption properties, atmospheric water harvesting, metal−organic frameworks, composites, stepped isotherm

## Abstract

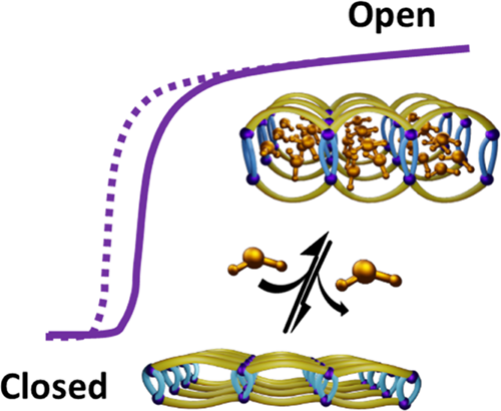

That physisorbents can reduce the energy footprint of water vapor capture and release has attracted interest because of potential applications such as moisture harvesting, dehumidification, and heat pumps. In this context, sorbents exhibiting an S-shaped single-step water sorption isotherm are desirable, most of which are structurally rigid sorbents that undergo pore-filling at low relative humidity (RH), ideally below 30% RH. Here, we report that a new flexible one-dimensional (1D) coordination network, [Cu(HQS)(TMBP)] (H_2_HQS = 8-hydroxyquinoline-5-sulfonic acid and TMBP = 4,4′-trimethylenedipyridine), exhibits at least five phases: two as-synthesized open phases, α ⊃ H_2_O and β ⊃ MeOH; an activated closed phase (γ); CO_2_ (δ ⊃ CO_2_) and C_2_H_2_ (ϵ ⊃ C_2_H_2_) loaded phases. The γ phase underwent a reversible structural transformation to α ⊃ H_2_O with a stepped sorption profile (Type F-IV) when exposed to water vapor at <30% RH at 300 K. The hydrolytic stability of [Cu(HQS)(TMBP)] was confirmed by powder X-ray diffraction (PXRD) after immersion in boiling water for 6 months. Temperature-humidity swing cycling measurements demonstrated that working capacity is retained for >100 cycles and only mild heating (<323 K) is required for regeneration. Unexpectedly, the kinetics of loading and unloading of [Cu(HQS)(TMBP)] compares favorably with well-studied rigid water sorbents such as Al-fumarate, MOF-303, and CAU-10-H. Furthermore, a polymer composite of [Cu(HQS)(TMBP)] was prepared and its water sorption retained its stepped profile and uptake capacity over multiple cycles.

## Introduction

**Figure 1:**
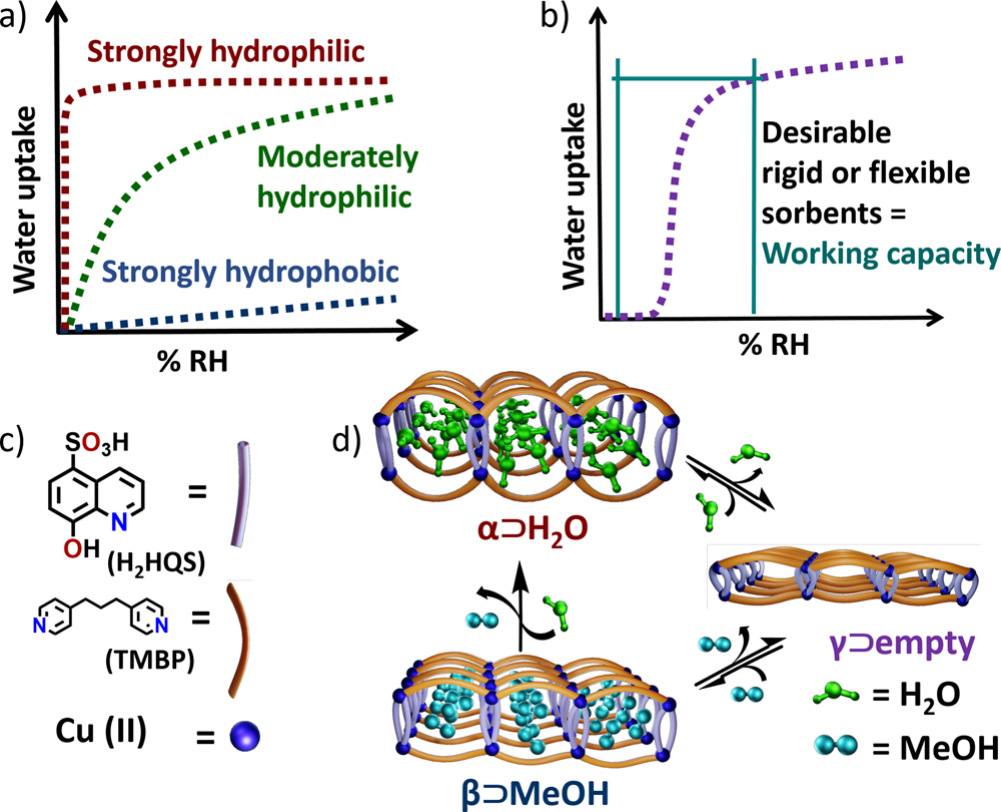
Illustration of water vapor sorption profiles and structural transformations in [Cu(HQS)(TMBP)] (H_2_HQS = 8-hydroxyquinoline-5-sulfonic acid and TMBP = 4,4′-trimethylenedipyridine). (a) Rigid sorbents typically exhibit strongly hydrophilic (red), moderately hydrophilic (green), or strongly hydrophobic (blue) isotherms. (b) Stepped sorption profiles are desirable because they can offer high working capacity. (c) Organic linkers and Cu cations form the one-dimensional (1D) coordination network [Cu(HQS)(TMBP)], (d), which undergoes reversible transformation between its α and γ phases when water is removed or adsorbed. Reversible transformation between β and γ occurred when MeOH was removed or adsorbed. β transformed to α by heating at 353 K in air.

The emergence of a new generation of porous physisorbents for gas/vapor capture and storage has captured the imagination of materials chemists as exemplified by the emergence of porous liquids^1^ and various classes of porous crystalline solids.^2^ In this context, water vapor capture and storage are at the forefront as more than 70% of humanity is currently suffering from lack of ready access to clean water. Furthermore, the situation is deteriorating due to water pollution, climate change, and population growth.^3^ In addition, whereas desalination of seawater can be implemented, it is energy-intensive and economically viable only in coastal areas due to added transportation costs. Atmospheric water harvesting (AWH) is a promising alternative technology. There are three primary approaches to AWH: (a) fog collection by means of large nets is inexpensive and low-maintenance but requires fog formation and is therefore usually restricted to mountainous or coastal areas; (b) cooling air below its dew point is energy-intensive and economically infeasible under dry conditions; (c) AWH *via* adsorption of atmospheric water vapor by a porous desiccant^4^ and release by applying a temperature and/or humidity swing process offers an energy-efficient approach that can in principle be implemented even in the most arid locations. Physisorbents also offer potential to provide more energy-efficient solutions for related applications such as indoor humidity control, natural gas dehydration, thermal battery management, and temperature regulation using adsorption-based heat pumps and chillers.^5^ In this regard, traditional desiccants such as zeolites are highly effective at water vapor capture, even at low relative humidity (RH), and can offer high uptake capacity, but their strong hydrophilicity typically requires high regeneration temperatures (Figure 1a). Porous physisorbents that exhibit S-shaped or stepped water sorption isotherms below 30% RH are more desirable as they can offer both high working capacity (Figure 1b) and low regeneration energy. Unfortunately, such water vapor isotherms remain rare amongst traditional desiccants.^6,7^

Metal–organic materials (MOMs),^8^ also known as porous coordination polymers (PCPs)^9,10^ or metal–organic frameworks (MOFs),^11,12^ crystalline materials formed by the assembly of metal ions/clusters and organic linkers, are promising candidates for AWH because their pore size/chemistry can be fine-tuned to optimize water sorption properties. Rigid MOF sorbents, as exemplified by MOF-801,^13,14^ UiO-66,^14^ CAU-10-H,^15,16^ MOF-303,^13,17,18^ Cr-soc-MOF-1,^19^ Co_2_Cl_2_BTDD,^20^ and Al-fumarate,^21^ have been studied with respect to their water sorption properties and offer desirable S-shaped isotherms^22−25^ thanks to a pore-filling mechanism. Alternatively, S-shaped or stepped isotherms can result from structural flexibility induced by exposure to gases or vapors.^26^ Flexible MOFs, also known as soft PCPs, undergo structural changes, sometimes dramatic, in response to external stimuli.^26−31^ Flexible sorbents that switch between closed and open phases are of special interest as they offer high working capacities and could find utility in gas and vapor storage applications.^32,33^ With respect to water sorption applications, such sorbents remain understudied.^34,35^ In this work, we report that a flexible 1D coordination network, [Cu(HQS)(TMBP)]·guest (guest = H_2_O, MeOH), transforms to a closed phase, γ, when activated by heat (Figure 1d). We herein report characterization of five crystal forms of [Cu(HQS)(TMBP)] and the water sorption performance of γ and its polymer composite.

## Experimental Section

All reagents were purchased in high-purity grade and used as received. More details are provided in the Supporting Information (SI).

### Preparation of [Cu(HQS)(TMBP)]·nH_2_O, α ⊃ H_2_O

In a typical reaction, a solution of water (2 mL) containing Cu(NO_3_)_2_·3H_2_O (48 mg, 0.20 mmol) was layered over a 5:2 solution of EtOH and water (7 mL) containing 8-hydroxyquinoline-5-sulfonic acid (H_2_HQS; 22.5 mg, 0.100 mmol) and 4,4′-trimethylenedipyridine (TMBP; 60 mg, 0.30 mmol). Then the reaction vessel was transferred into an oven and heated at 333 K for one day, at which point green single crystals were harvested from the wall and bottom of the vessel. A higher yield of crystals was obtained after standing for 2–3 days.

### Preparation of [Cu(HQS)(TMBP)]·nMeOH, β ⊃ MeOH

A solution of Cu(NO_3_)_2_·3H_2_O in 2 mL of water was layered over a MeOH (5 mL) solution of H_2_HQS and TMBP. The procedure, including reaction conditions and concentrations, was the same as above.

### Preparation of [Cu(HQS)(TMBP)], γ

α ⊃ H_2_O was activated at 353 K under vacuum for several hours to transform into γ. Similarly, β ⊃ MeOH was activated at 353 K under vacuum to afford γ.

### Preparation of [Cu(HQS)(TMBP)]–Polymer Composite

A powdered sample of [Cu(HQS)(TMBP)] (0.55 g), HYCAR 26410 polymer binder (0.5 g), isopropanol (3.85 g), and water (9.7 g) were mixed in a beaker and stirred using a mechanical mixer. The resulting slurry was placed on a Teflon plate and heated in an oven at 393 K for 1 h.

## Results and Discussion

### Synthesis and Structural Characterization

**Figure 2:**
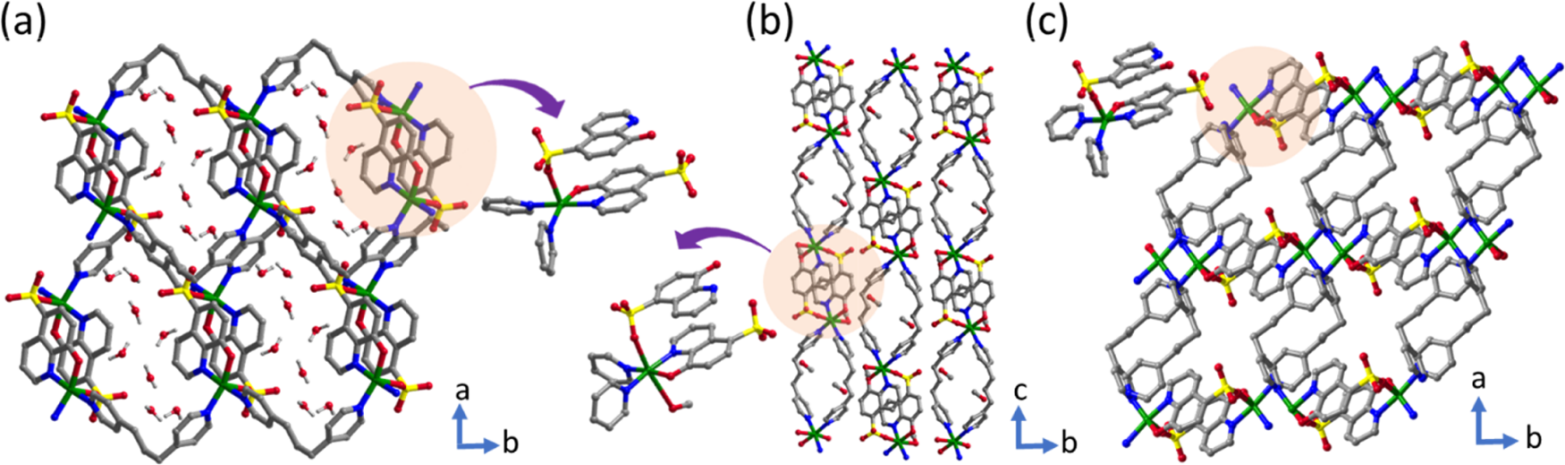
Crystal structures of the as-synthesized (α, β) and activated (γ) phases. (a) In α, HQS coordinates to metal centers in parallel but oriented in opposite directions and, when connected by TMBP linkers, formed a 1D coordination with water channels. (b) In β, metal cations coordinate to MeOH and TMBP linkers to form a 1D structure that packs to form channels occupied by MeOH. (c) γ is the phase obtained upon activation; the TMBP linkers are squeezed, resulting in reduced guest accessible space.

Dark green single crystals of α ⊃ H_2_O were isolated as described above (Figure S1). Single-crystal X-ray diffraction (SCXRD) analysis revealed that α ⊃ H_2_O had crystallized in the triclinic space group *P*1̅ (Table S1). Square pyramidal Cu cations coordinate to an axial sulfonate oxygen atom, whereas the equatorial positions are occupied by an HQS ligand (oxygen of oxo moiety and nitrogen of pyridine moiety) and two nitrogen atoms of two different TMBP ligands (Figure 2a). Adjacent Cu cations are connected by TMBP linker ligands to form cavities linked by HQS anions to generate a 1D coordination polymer (Figure 2a). Four independent water molecules were found to be present in the channels that result from eclipsed alignment of cavities. The use of MeOH/water as the solvent system for synthesis afforded a different phase, β ⊃ MeOH. SCXRD revealed that β ⊃ MeOH had also crystallized in *P*1̅ (Table S1) with the same connectivity as α ⊃ H_2_O, except that one MeOH coordinates to what is now an octahedral Cu cation (Figure 2b). The α and β phases exhibit accessible void spaces of 16.0 and 18.3% of unit cell volume, respectively (Figures S2 and S3). Phase purity of the as-synthesized phases was confirmed by powder X-ray diffraction (PXRD, Figure S4). Dehydration of α ⊃ H_2_O and desolvation of β ⊃ MeOH by heating at 353 K *in vacuo* resulted in transformation to a closed phase, γ (Figures 1d, S4 and S5). We also observed that β ⊃ MeOH transformed to α ⊃ H_2_O when heated at 353 K in ambient air (Figure S4). [Cu(HQS)(TMBP)] retained crystallinity after soaking in water at RT or in boiling water for at least 6 months (Figure S6). Thermogravimetric analysis (TGA) indicated that both α ⊃ H_2_O and β ⊃ MeOH are thermally stable in N_2_ flow to 503 K with ∼7.5% weight loss corresponding to 4 H_2_O or 3 MeOH molecules per formula unit, respectively (Figures S10 and S11). Cu-based MOFs such as HKUST-1 are known to suffer from poor hydrolytic stability. We attribute the excellent thermal and hydrolytic stability of [Cu(HQS)(TMBP)] to two factors: (i) chelation by HQS linkers and (ii) that Cu cations are not exposed to the pore surface in both the α and γ phases. Single crystals of α ⊃ H_2_O and β ⊃ MeOH converted to microcrystalline powders of γ upon activation. We collected high-resolution synchrotron PXRD data of a sample of γ and determined its crystal structure by Rietveld refinement (Figure S12). γ retained the network connectivity of the as-synthesized phases, but its void space was reduced to 7.5% of unit cell volume (Figure S13). The experimental and calculated PXRD patterns of γ match well (Figure S4).

### Gas Sorption and *In Situ* PXRD Studies

**Figure 3:**
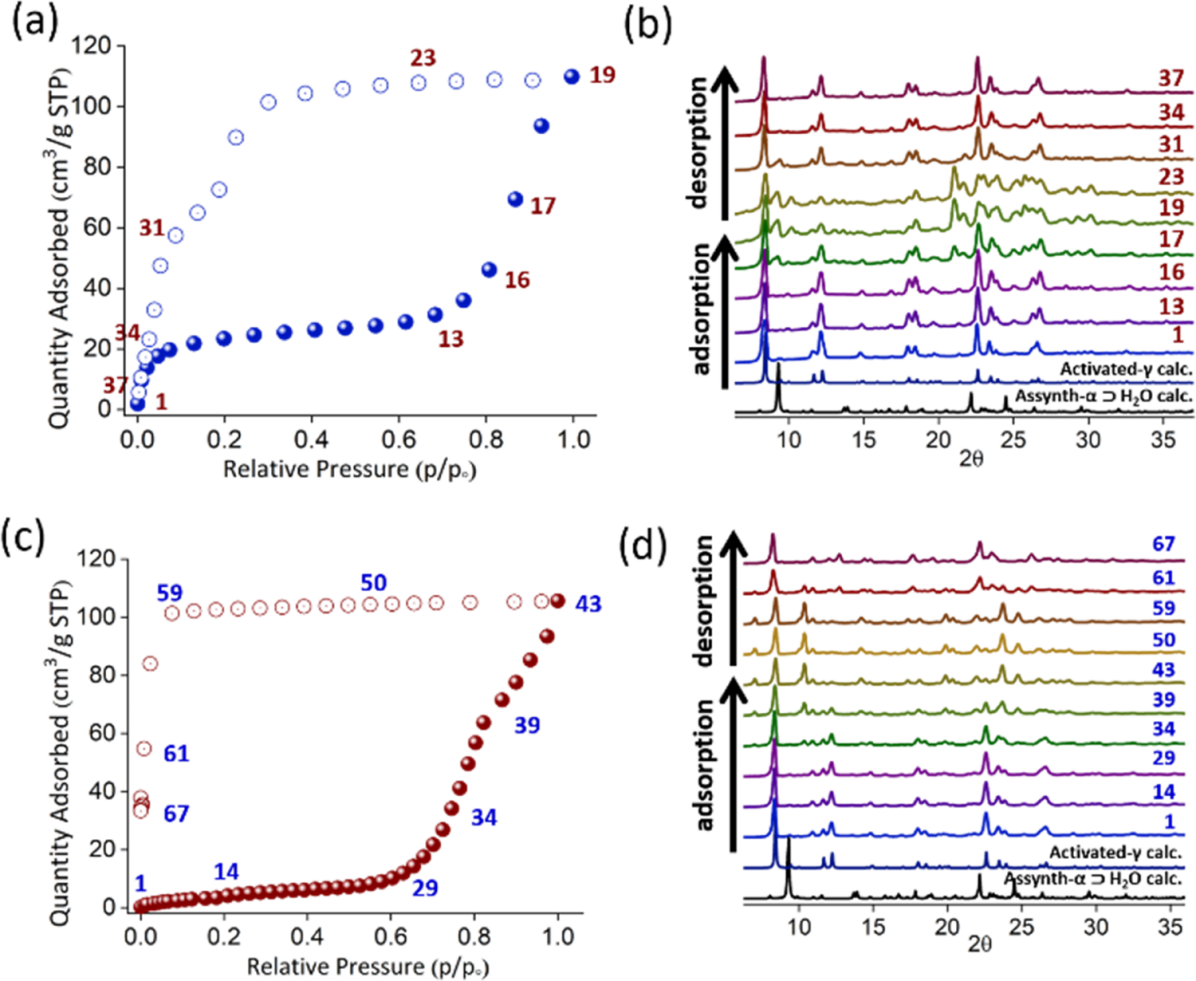
*In situ* coincident PXRD measurements for CO_2_ and C_2_H_2_ at 195 K. CO_2_ sorption isotherm (a) with selected PXRD patterns plotted in (b). C_2_H_2_ sorption isotherm (c) with selected PXRD patterns plotted in (d).

Motivated by the structural flexibility that accompanied loss of guest molecules, we collected the CO_2_ and C_2_H_2_ sorption isotherms of [Cu(HQS)(TMBP)] at 195 K (Figure 3a,c), following activation of α ⊃ H_2_O at 353 K under vacuum for 12 h. A stepped isotherm was obtained for CO_2_ (Figure 3a). In the low-pressure region from *P*/*P*_0_ = 0.1 to 0.7, the uptake reached 30 cm^3^/g before the step to an uptake of 110 cm^3^/g at *P*/*P*_0_ = 1. CO_2_ desorption does not match adsorption because of hysteresis, the reverse step being at *P*/*P*_0_ = 0.2. For C_2_H_2_ (Figure 3c), gradual uptake from 0 to 10 cm^3^/g occurred before a step at *P*/*P*_0_ = 0.65 (gate-opening pressure) that resulted in an uptake of 110 cm^3^/g at *P*/*P*_0_ = 1. C_2_H_2_ desorption also exhibited hysteresis.

To gain insight into the structural transformations associated with the stepped sorption isotherms, we conducted *in situ* coincident PXRD measurements with CO_2_ and C_2_H_2_ at 195 K (Figure 3b,d). Representative PXRD patterns corresponding to points on the adsorption and desorption profiles were plotted. CO_2_ sorption revealed that, from points 1 to 16, γ remained present as the experimental PXRD pattern matched its calculated PXRD pattern (Figure 3b). Increasing pressure afforded new PXRD peaks, which we attribute to a structural transformation from γ to a CO_2_-loaded phase (δ ⊃ CO_2_). δ ⊃ CO_2_ remained present after further increases in pressure (17 to 19) and pressure reduction (23 to 31). Further reduction in pressure resulted in transformation back to γ (34 and 37). With respect to *in situ* C_2_H_2_ sorption (Figure 3d), the PXRD data revealed that γ reversibly transformed to a C_2_H_2_-loaded phase (ϵ ⊃ C_2_H_2_). From points 1 to 34, γ was unchanged. Increasing pressure to point 43 (adsorption) afforded ϵ ⊃ C_2_H_2_ as indicated by PXRD. This phase remained until pressure was decreased at point 59, below which desorption occurred. The PXRD pattern at point 67 corresponds to γ. Both the CO_2_ and C_2_H_2_ pure gas isotherms exhibit large hysteresis, which is undesirable from a practical gas storage perspective.

### Water Vapor Sorption

**Figure 4:**
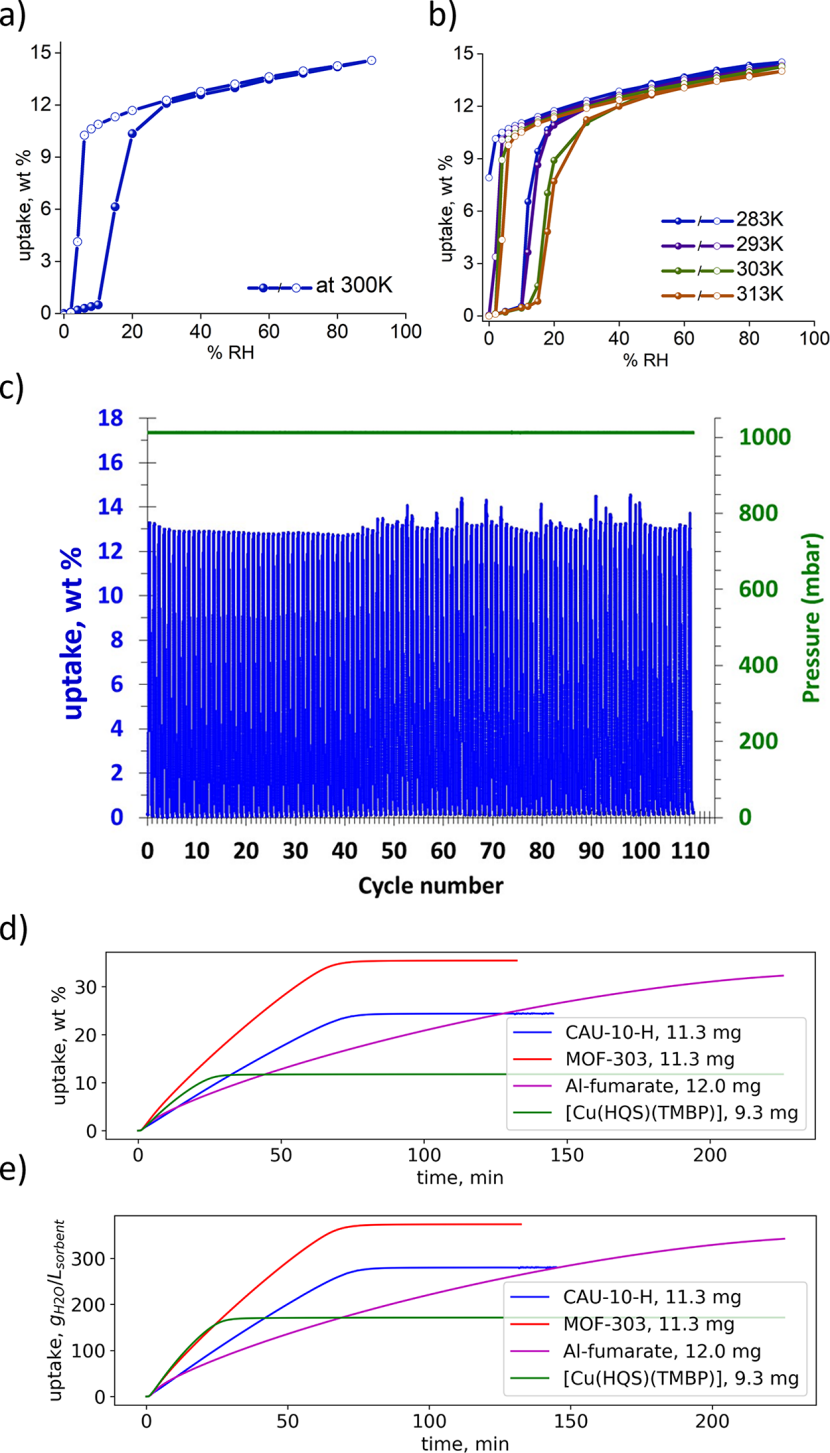
Water vapor sorption studies on [Cu(HQS)(TMBP)]. Water vapor sorption isotherms at 300 K (a) and 283, 293, 303, and 313 K (b) measured using an intrinsic dynamic vapor sorption (DVS) instrument. (c) Water vapor temperature-humidity swing cycling (300 K, 60% RH to 322 K, 0% RH) measured using an intelligent gravimetric analyzer (IGA). (d, e) Comparison of the kinetics of water loading on [Cu(HQS)(TMBP)] with three leading rigid MOF sorbents—MOF-303, CAU-10-H, and Al-fumarate—when subjected to humidity swing cycling (30% RH at 300 K to 0% RH at 300 K).

The presence of water molecules in α ⊃ H_2_O and its structural transformation upon water removal prompted us to study the water vapor sorption properties of [Cu(HQS)(TMBP)] at 300 K. Interestingly, γ exhibited a single-step adsorption profile (Type F-IV) at low RH with relatively small hysteresis. Whereas negligible uptake was observed up to 10% RH, uptake increased to 12 wt % at 30% RH followed by a gradual increase to 15 wt % at 90% RH. The desorption profile revealed the relatively small hysteresis, complete desorption occurring by 6% RH (Figure 4a). Water sorption isotherms measured at 283, 293, 303, and 313 revealed gate-opening pressures of 10, 10, 12, and 15% RH, respectively (Figure 4b). The gate-closing pressures were observed to be similar for each temperature except for the isotherm measured at 283 K. No difference in water sorption properties was observed for γ prepared from α ⊃ H_2_O *vs* that prepared from β ⊃ MeOH (Figure S17). To confirm that a phase transformation had indeed occurred, we conducted *in situ* PXRD measurements. The resulting PXRD patterns indicate that exposure to water vapor had induced transformation of γ to α ⊃ H_2_O (Figure S16).

To verify recyclability, we conducted vacuum swing cycling experiments (60% RH at 300 K to 0% RH at 300 K, Figure S27). After 70+ cycles, the working capacity was unchanged at 13.2 wt %, matching the uptake from the sorption isotherm and indicating potential utility for water vapor applications. Temperature-humidity swing cycling experiments were conducted to further evaluate sorbent performance and stability. In a typical experiment, we allowed 8.47 mg of [Cu(HQS)(TMBP)] to adsorb for 14 min at 300 K (60% RH) and desorb for 20 min at 322 K (0% RH). More than 100 cycles were collected, and they reveal that the working capacity of 12.5 wt % was retained (Figure 4c).

Whereas flexible sorbents have the potential to improve working capacity and mitigate the effects of heat release during adsorption,^33,36^ their relative kinetics remains understudied. Slow kinetics of adsorption or/and desorption on rigid sorbents will necessarily reduce performance for AWH applications unless day/night cycling is being considered.^20^ Intuitively, one might expect that phase changes would also slow the kinetics of adsorption/desorption in a structurally flexible sorbent. Indeed, structural changes in CAU-15-Cit resulted in slow adsorption kinetics in comparison to its more rigid analogue, CAU-10-H.^37^ Unexpectedly, we observed that the kinetics of water loading and unloading for [Cu(HQS)(TMBP)] are relatively fast. Indeed, [Cu(HQS)(TMBP)] compares favorably with the kinetics of three well-studied rigid MOF water sorbents, MOF-303, CAU-10-H, and Al-fumarate (Figures 4d and S25), particularly when volumetric uptake is considered (Figures 4e and S25). To measure kinetics of loading and unloading, 50–100 μm sieved fractions of the rigid sorbents were prepared. The mean particle size of [Cu(HQS)(TMBP)] was determined to be *ca*. 5 μm (Figures S14–S15), precluding a direct comparison. That the structural transformations in [Cu(HQS)(TMBP)] do not slow kinetics under the tested conditions should promote a more comprehensive study of sorption kinetics in flexible sorbents.

For industrial applications, the use of microcrystalline powders poses technical challenges, and we therefore prepared a polymer composite of microcrystalline [Cu(HQS)(TMBP)] and studied its properties. Mixing of the powder and polymer binder solution in a 50:50 weight ratio and curing the resulting slurry for 1 h at 393 K (Figure S28) resulted in formation of a composite. That [Cu(HQS)(TMBP)] survives the curing process and is present in the composite was confirmed by PXRD (Figure S30). The water vapor sorption isotherm of the composite measured at 300 K retained the stepped profile of pure [Cu(HQS)(TMBP)] (Figure S33) and is the weighted sum of individual [Cu(HQS)(TMBP)] powder and pure polymer isotherms (Figure S35), the polymer content being less than 50% of the composite by weight because of binder solutionmass loss. Zero to sixty percent RH vacuum swing cycling tests were conducted and revealed that a working capacity of 11.5 wt % was maintained for >240 cycles (Figure S36).

### Structural Insights

To better understand the nature of host–guest interactions and the structural transformations, we analyzed the crystal structures of α ⊃ H_2_O, β ⊃ MeOH, and γ. In each structure, two HQS linkers lie in parallel but oriented in opposite directions and exhibit π···π stacking interactions, *D*_C···C_ = 3.615(5), *D*_C···C_ = 3.503(1), and *D*_C···C_ = 3.435 Å, respectively (Figures S37, S39 and S40). In α ⊃ H_2_O, square water clusters are sustained by O–H···O hydrogen bonds (*D*_OH···O_ = 2.76 and 2.79 Å, Figure S38). The water tetramer is H-bonded to four water molecules (*D*_OH···O_ = 2.73 and 2.94 Å) forming an octameric water cluster around a center of inversion. As revealed by Figure S38, the peripheral water molecules form charge-assisted H-bonds with framework functional groups as follows: bridging between two sulfonate oxygen atoms (*D*_OH···O_ = 2.75 and 2.90 Å); phenolate oxygen atom (*D*_OH···O_ = 2.78 Å). The resulting stoichiometry, four water molecules per formula unit, is consistent with the observed uptake capacity and TGA data. With respect to β ⊃ MeOH (Figure S39), each MeOH molecule forms a charge-assisted H-bond with a sulfonate moiety (*D*_OH···O_ = 2.76 Å). The TMBP linker is in effect stretched (∠C–C–C = 109.2 to 112.8°) instead of slightly bent in α ⊃ H_2_O (∠C–C–C = 110.7 to 115.8°). In the case of γ (Figure S40), the alkane chain of TMBP linkers is even more stretched (∠C–C–C = 85.98 to 108.3°). The metal-to-metal distances within and between 1D chains are shorter than those in the α and β phases, *D*_M···M_ = 11.414(123), 7.503(119), and 4.682(134) Å, respectively. These results indicate that γ underwent large distortions following guest removal. The low-angle PXRD peaks and corresponding lattice planes most impacted by the phase transformation between the α and γ forms are illustrated in Figures S41 and S42, respectively.

## Conclusions

A new flexible coordination network, [Cu(HQS)(TMBP)], was found to exhibit switching between closed and open phases with multiple sorbates including water vapor. Sorption of water vapor at 300 K induced structural transformation with a single-step isotherm at low RH and small hysteresis, whereas sorption of gases (CO_2_, C_2_H_2_) at 195 K induced stepped isotherms with large hysteresis. The hydrolytic stability of [Cu(HQS)(TMBP)] is high, being unaffected after soaking in boiling water for >6 months and following multiple water vapor adsorption/desorption cycles. Interestingly, the kinetics of water loading and unloading compare favorably with leading rigid water sorbents such as MOF-303, Al-fumarate, and CAU-10-H. Furthermore, [Cu(HQS)(TMBP)] can be formulated into a polymer composite, and its characteristic single-step water vapor sorption isotherm was retained. Overall, this work reveals that flexible 1D coordination polymers can offer optimal kinetics and thermodynamics for water sorption and could be further explored as candidates in the context of AWH and related applications.

## Supporting Information Available

Material synthesis and characterization methods, crystallographic parameters and crystal structure figures for all phases, PXRD patterns, TGA, SEM images, water vapor sorption isotherms and kinetic plots, sorption cycling plots (PDF)
