# Supplementary material for: Flexible Coordination Network Exhibiting Water Vapor–Induced Reversible Switching between Closed and Open Phases
Source: ACS Appl Mater Interfaces. 2022 Aug 17;14(34):39560–6. doi: 10.1021/acsami.2c10002 (PMC9437871; doi:10.1021/acsami.2c10002)
Supplement: Supplementary file 1 — am2c10002_si_001.pdf [file am2c10002_si_001.pdf]

Supporting Information

Flexible Coordination Network Exhibiting Water Vapor-Induced Reversible Switching between Closed and Open Phases

Mohana Shivanna,[‡a,b] Andrey A. Bezrukov,[‡a] Victoria Gascón-Pérez,[a] Kenichi Otake,[b] Suresh Sanda,[a] Daniel J. O'Hearn,[a] Qing-Yuan Yang,[a] Susumu Kitagawa*[b] and Michael J. Zaworotko*[a]

[a] Dr. M. Shivanna, Dr. A. A. Bezrukov, Dr. V. Gascón-Pérez, Dr. S. Sanda, Mr. D. J. O'Hearn, Dr. Q-Y, Yang, Prof. M. J. Zaworotko
Department of Chemical Sciences, Bernal Institute, University of Limerick, Limerick V94 T9PX, Republic of Ireland.
E-mail: Michael.Zaworotko@ul.ie

[b] Dr. M. Shivanna, Dr. K. Otake, Prof. S. Kitagawa
Institute for Integrated Cell-Material Sciences, Kyoto University Institute for Advanced Study, Kyoto University Yoshida Ushinomiya-cho, Sakyo-ku, Kyoto 606-8501, Japan.

‡ These authors contributed equally.

Table of Contents

1.	Materials and methods	S03-5
2.	Crystallographic parameters	S06
3.	As synthesized single crystals and crystal structure	S07-9
4.	PXRD comparisons	S10-15
5.	Thermogravimetric analysis (TGA)	S16-17
6.	PXRD refinement	S18
7.	Activated crystal structure and particle size measurements	S19-21
8.	Water vapor sorption isotherms and in-situ PXRD	S22
9.	Flexible sorbents for water vapor sorption	S23
10.	Water vapor sorption at different temperatures	S24
11.	Water vapor sorption for reported sorbents	S25-27
12.	Kinetics and water sorption cycles	S28-34
13.	Composite preparation, characterization and water vapor sorption	S35-41
14.	Water vapor sorption cycling on composite	S42
15.	Crystal structure analysis	S43-48
16.	References	S49

1. Materials and Methods

Synthesis of Al(OH)Fumarate, [[Al(OH)(fumarate)]·nH_2O]$_n$

Al(OH)Fumarate was synthesized following a previously reported synthesis method at a smaller scale compared to the reported scale.[1] In a 100 mL round bottom flask, $AlCl_3·6H_2O$ (2.69 g, 10.8 mmol) and fumaric acid (1.54 g, 13.3 mmol) were dissolved in 55 mL of DMF. The resulting turbid, yellowish mixture was stirred at 130 °C for 4 days. After cooling, a snow-white precipitate was filtered off and stirred twice in acetone (1 h and 24 h, 20 mL each), and twice in ethanol (1 h and 24 h, 20 mL each). The solid was dried at 80 °C. Yield: 1.54 g, 90%. Activation of Al(OH)Fumarate was carried out under vacuum at 90 °C for 8 hours.

Synthesis of CAU-10-H [[Al(OH)(benzene-1,3-dicarboxylate)]·nSolvent]$_n$

CAU-10-H was synthesized and activated using a previously reported synthesis method.[2] A mixture of $Al_2(SO_4)_3·18H_2O$ (800 mg, 1.20 mmol), benzene-1,3-dicarboxylic acid (200 mg, 1.20 mmol), 1 mL of DMF, and 4 mL of H_2O were added to a Teflon-lined steel-autoclave with a volume of 22.5 mL which were placed in an oven preheated at 135 °C for 12 h. The product was filtered off when temperature cooled down to room temperature. The filtered solid was re-dispersed in water by sonication and stirring, until a homogeneous mixture was obtained. The dispersion was filtered again and the white microcrystalline solid was dried in air. Yield, 230 mg, 91%. Activation of CAU-10-H was carried out under vacuum at 200 °C for 12 hours.

Synthesis of [Al(OH)(1H-pyrazole-3,5-dicarboxylate)], MOF-303

MOF-303 was synthesized using a previously reported synthesis method.[3] 3,5-pyrazoledicarboxylic acid monohydrate (3.75 g, 21.55 mmol) was dissolved in deionized H2O (362.5 mL) and LiOH solution (2.57 M, 12.5 mL). The resulting solution was heated for 30 min in a pre-heated oven at 120 °C. Afterwards, $AlCl_3·6H_2O$ (5.2 g, 21.55 mmol) was added to the solution. Any precipitate was dissolved under sonication and vigorous shaking. Once a clear solution was obtained, the hot reaction mixture was put in a pre-heated oven at 100 °C, where it was kept for 15 hours. The obtained precipitate was filtered out and washed with water. The snow-white solid was subsequently washed with anhydrous MeOH for 24 hours in a Soxhlet apparatus and air-dried for 3 days. Activation of the MOF-303 was carried out under vacuum at 150 °C for 12 hours.

PXRD measurements and analysis

In-house powder X-ray diffraction (PXRD) patterns were measured on a Rigaku SmartLab X-ray diffractometer using Cu−K α radiation (λ = 1.54178 Å). For each measurement we placed a few mg of powder sample on a glass substrate and measured angle (2θ) from 5° to 40° with 0.02° increment.

To determine the γ⊃empty phase we collected synchrotron PXRD data using synchrotron X-ray (λ = 0.80000 Å) and multiple MYTHEN detectors of the BL02B2 beamline at Super Photon ring (SPring-8). Unit cell parameters of activated phase were found using indexing with DICVOL algorithm implemented in DASH.[4] Structure determination of activated phase was conducted using GSAS-II.[5] Structure of activated phase was solved using Monte Carlo-Simulated Annealing. A Pawley refinement was used to obtain structure factors. Solution was found using three rigid bodies generated from the structure of as-synthesized phase: Cu-coordinated 8-hydroxy-5-quinolinesulfonic anion and two 4-methylpyridine moieties. The structure solution found using Monte Carlo-Simulated Annealing was further refined using Rietveld refinement. Positions of Cu^{2+} were freely refined, while positions of atoms belonging to the organic ligands were refined with angle, bond length and plane restraints applied. Rietveld refinement plot can be found in Figure S12.

SC-XRD measurements

Suitable single crystals of as-synthesised α⊃H_2O and β⊃MeOH were selected for X-ray diffraction. Intensities were collected on a Rigaku XtaLAB AFC10 diffractometer using a VariMax Mo Optic with Mo-Kα (λ = 0.71073 Å). These structures were solved by direct methods and refined on F2 by full-matrix least-squares methods with SHELXTL version 2018/3.[6]

Supplementary crystallographic data for this manuscript has been deposited at the Cambridge Crystallographic Data Centre under deposition numbers CCDC 2168033, 2168034, and 2167897.

Coincident PXRD sorption measurements

Coincident PXRD sorption measurements were conducted using Rigaku Smartlab with CuKα radiation (Rigaku, Japan) connected to a BELSORP-18PLUS volumetric adsorption equipment (MicrotracBEL Japan, Corp.). Cryo-system was connected to control the required temperature. At first the as-synthesized sample was activated under a high vacuum (at a pressure around 200 Pa) at 80 °C for 12 h. Then the activated sample transferred into the in-situ chamber and activated again for 2 h at 80 °C in order to complete removal of moisture. C_2H_2 and CO_2 adsorption was carried out at -78 °C by controlling the temperature using the cryostat. The *in-situ* PXRD pattern was measured at each adsorption and desorption equilibrium point.

TGA measurements

The TGA measurements were carried out using a Rigaku TG 8120 analyzer (EVO2 TG/S-SL) at 5 °C min^{-1} heating rate under N_2 atmosphere.

Vacuum Dynamic Vapor Sorption (DVS) measurements

Vacuum dynamic vapor sorption (DVS) measurements were conducted using a Surface Measurement System DVS Vacuum device (UK). The DVS instrument used for these studies measures the uptake and loss of water vapor gravimetrically. Ex-situ activated samples were further in-situ degassed under high vacuum pressure (up to $2 \cdot 10^{-6}$ Torr) to establish the dry mass. Water adsorption/desorption cycles were used for the determination of recyclability and working capacity (wt. % units) of sorbent. The recycling tests were performed in a temperature-controlled incubator at 27 °C. For each material, 70-170 cycles of adsorption (0→60% P/P_0, 10 min) followed by desorption (60→0% P/P_0, 10 min) were conducted at 27 °C. The mass of every sample was determined by comparison with an empty reference pan and recorded by a high-resolution microbalance with a mass resolution (precision) of ± 0.1 μg. The high mass resolution and its excellent baseline stability allow the instrument to measure the adsorption and desorption of small amounts of water molecules. The vapor partial pressure around the sample is controlled by mixing saturated and dry carrier vapor streams using electronic mass flow controllers. The temperature was kept constant at ± 0.2 °C accuracy by enclosing the entire system in a temperature-controlled incubator. Pure water (HPLC Gradient Grade, Fisher Chemical) was used as the adsorbate for the recycling studies.

Intrinsic Dynamic Vapor Sorption (DVS) Measurements

Water vapor adsorption–desorption experiments at atmospheric pressure were performed using a dynamic vapor sorption (DVS) intrinsic analyzer (from Surface Measurement Systems, UK). The DVS Intrinsic is designed to accurately measure sample's mass change as it sorbs high-precisely controlled concentrations of water vapor using air as a carrier gas. The sample (ca. 5-10 mg) was loaded into a stainless-steel pan and suspended from an SMS ultra-sensitive recording microbalance (with a resolution of 0.1 μg) with the help of a hang-down wire. Before the experiment started, once the sample was loaded from said hang-down wire, it was allowed to reach temperature and humidity equilibria and baseline stability within its chamber for an abbreviated period of time. The sample was exposed to an air flow with known % RH (from 0 % RH to 90 % RH) in the adsorption/desorption branches, respectively. The flow-rate used in the experiments was 200 sccm (standard cubic centimeters per minute), and the temperatures employed were 298, 300, 303, 308 and 313 K (± 0.2 K to each). Equilibria determination of the sample mass at each RH stage was performed by measuring the change rate percentage of mass over time (dm(%)/dt(min)). Not until said dm/dt reached a value of 0.002 % min^{-1}, the equilibria were achieved (with an accuracy of ± 1.0 % RH), and the device was allowed to measure the next stage of RH. So, the sample mass readings obtained from the microbalance within said equilibria revealed the vapor adsorption/desorption behavior of the sample. Consequently, isotherm analysis and kinetics profiles of water vapor sorption and desorption were recorded.

Intelligent Gravimetric Analyzer (IGA) Measurements

Water vapor adsorption–desorption experiments at atmospheric pressure (1013 mbar, 760 torr) were performed using a IGAsorp dynamic vapor sorption (DVS) analyzer from Hiden Isochema Ltd., UK. The IGAsorp system uses the gravimetric method, which provides an correct determination of the sorption and interaction of water vapor with the material. The ultra-sensitive microbalance accurately measures the water uptake/loss as the programmed conditions are changing (resolution 0.1 μg, stability ± 1 μg). This microbalance is independently thermostated to keep calibration, enhance weighing stability and anti-condensation protection. The sample reactor, humidity sensor and humidifier are housed in a

single machined block of aluminum, thus minimizing temperature gradients. This block is thermostated by pumping fluid into an internal circulation circuit from an external high stability water bath with computerized feedback control and thermostat, to keep temperature uniformity of the critical components (regulation accuracy ± 0.05 K). Additionally, a programmable heater is fitted internally to the sample reactor and used for precisely controlled in-situ sample drying. The heater enables temperatures up to 350 °C to be achieved at ramp rates up to 10 °C/min. The temperature sensor, a platinum resistance thermometer (Pt100), allows a measurement accuracy of ± 0.1 K. About the vapor generation, a split flow technique in conjunction with computerized feedback control system is used to regulate humidity in the sample chamber. The rapid response relative humidity (RH) sensor and accurate mass flow controllers (± 1 %RH) ensure maintain a flow of gas of a defined humidity over the sample.

Prior to the experiment, the sample was allowed to reach temperature and humidity equilibria within the chamber for a specific period of time. The sample was exposed to desired % RH (60 or 0% RH) and temperatures in the adsorption (27 °C) /desorption (49 °C) branches, respectively.

Adventure Dynamic Vapour Sorption (DVS) Measurements

Water vapour sorption was performed using Adventure dynamic vapour sorption (DVS) instrument manufactured by Surface Measurement Systems. The instrument gravimetrically measures water vapour uptake using air as a carrier gas. Digital mass flow controllers regulate flows of dry and saturated gases. Relative humidity is generated by precisely mixing dry and saturated gas flows in desired flow ratios which produce expected relative humidity. Pure water was used to generate water vapour for these measurements and temperature was maintained at 300 K by enclosing the system in a temperature-controlled incubator. The mass of the sample was determined by a high resolution microbalance with a precision of 0.1 μg. Microbalance has symmetric configuration with two branches of the balance being exposed to the same gas and being kept at the same temperature, which allows negation of buoyancy and drag effects. Instrument allows measurement of 2 samples in parallel. Prior to the measurement, each sample was in-situ activated in dry air at 80-100 °C for 60 minutes using built-in preheater and consequently cooled to sorption temperature in 90 minutes.

Isotherm measurements were performed on approximately 10 mg of sample powder. 400 sccm/min flow was used for the measurements at 300 K. The flow is split between two samples. For each isotherm point, dm/dt < 0.05 %/min was used as criteria of reaching equilibrium.

Kinetics measurements were performed using a specified mass of activated sample. For rigid materials, kinetic measurements were performed on 50-100 μm sample fraction obtained by sieving. 400 sccm/min flow was used for the measurements, which is split between two samples. For adsorption and desorption kinetic curves, dm/dt < 0.05 %/min was used as criteria of reaching equilibrium.

Volumetric kinetics was calculated using following crystal densities of materials (g/cm^3): CAU-10-H: 1.148; MOF-303: 1.055; Al-fumarate: 1.060; [Cu(HQS)(TMBP)]: 1.457.

2. Crystallographic parameters

Table S1. Crystallographic data and structural refinement parameters.

	**As-synthesised α⊃H_2O**	**As-synthesised β⊃MeOH**	**Activated gamma** (γ)
Method	SC	SC	powder
Formula[a]	**$C_{22}H_{28}N_3O_8SCu$**	**$C_{24}H_{27}N_3O_6SCu$**	**$C_{22}H_{13}N_3O_4SCu$**
cF.W.	557.08	549.10	478.97
T (K)	100K	100K	298 K
Space group	$P\bar{1}$	$P\bar{1}$	$P\bar{1}$
a (Å)	10.453(2)	8.154(1)	11.4134(19)
b (Å)	11.495(2)	11.204(2)	10.5017(17)
c (Å)	12.129(2)	14.258(3)	10.2982(11)
α (°)	112.152(2)	101.18(2)	74.034(4)
β (°)	92.300(2)	93.075(2)	96.460(4)
γ (°)	112.983(2)	107.544(2)	113.120(4)
V (Å^3)	1212.34(5)	1209.8(4)	1091.40(4)
Z	2	2	2
D_c (g cm^{-3})	1.526	1.507	1.4575
μ (mm^{-1})	1.039	1.035	
Data collected/unique	6887/7116	7062/6599	
R_1 (>2σ)	0.0261	0.0288	
wR_2 (>2σ)	0.0717	0.0801	
wRp			0.08549
GOF	1.049	1.071	
CCDC number	2168033	2168034	2167897

3. As-synthesized single crystals and crystal structure

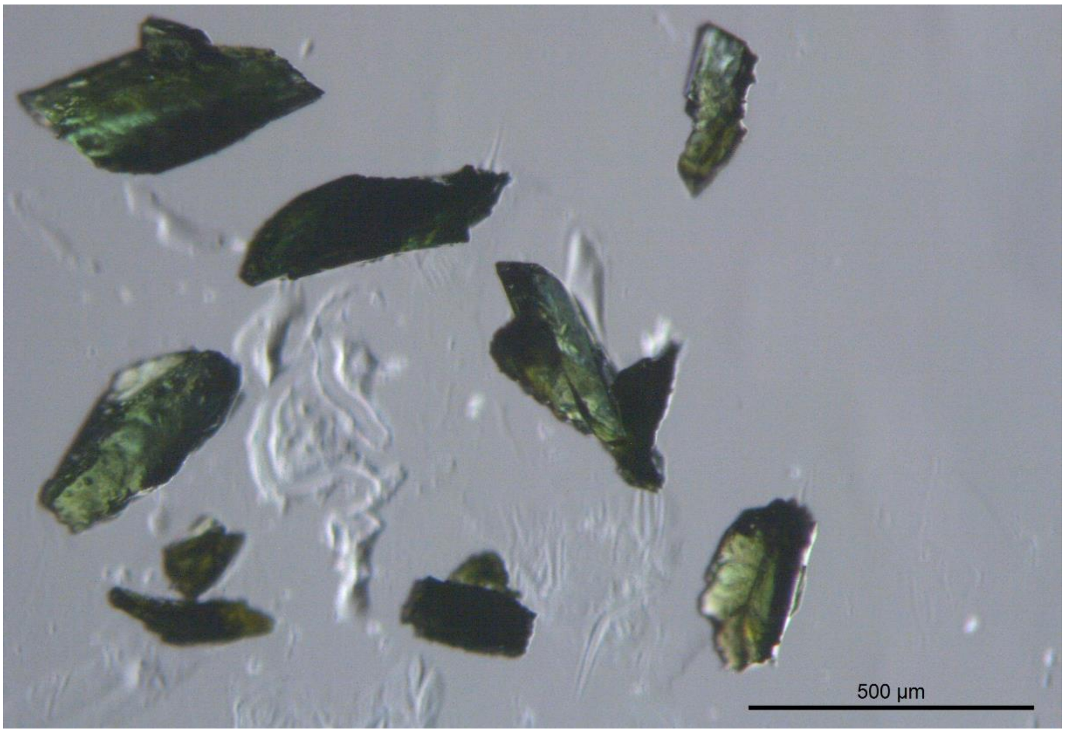

Figure S1. Microscopic images of as-synthesised single crystals of α⊃H_2O.

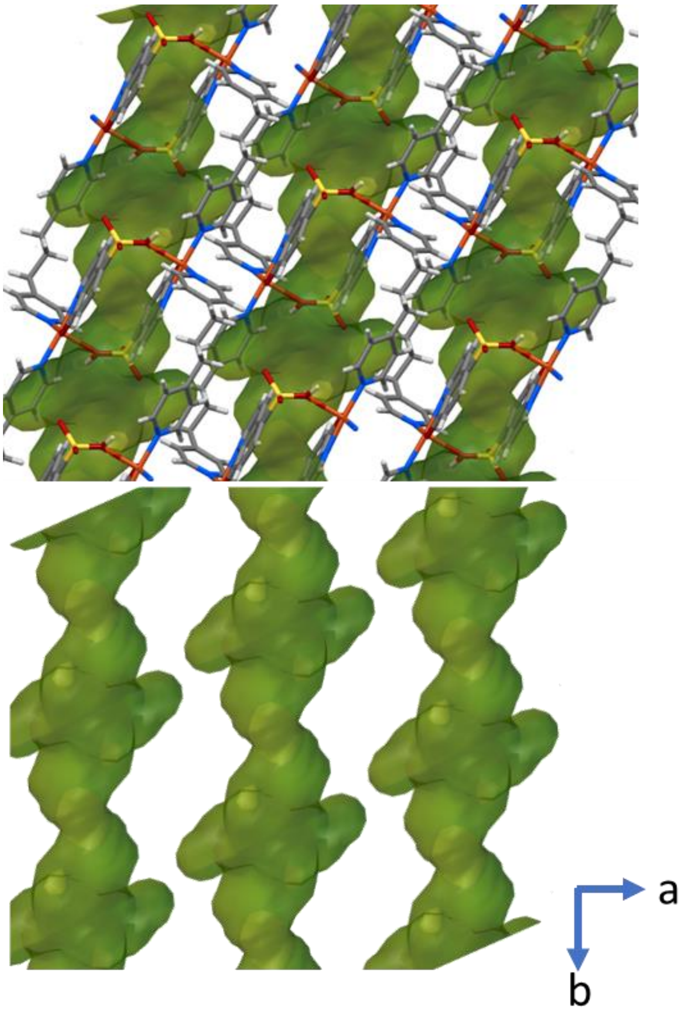

Figure S2. Closed packed structure of α⊃H_2O phase and representation of 1D guest accessible channels. The void space found to be 16.0% calculated by considering the probe radius of 1.2 Å and grid spacing of 0.7 Å.

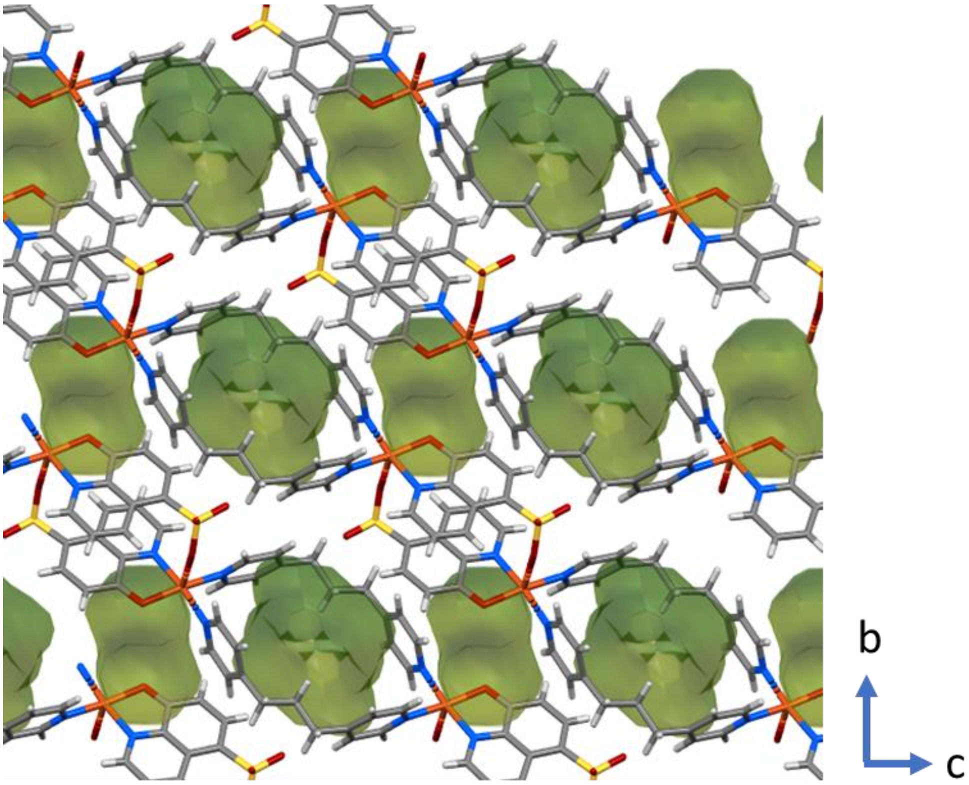

Figure S3. Closed packed structure of β⊃MeOH phase and representation of 0D guest accessible channels. The void space found to be 18.3% calculated by considering the probe radius of 1.2 Å and grid spacing of 0.7 Å.

4. PXRD comparisons

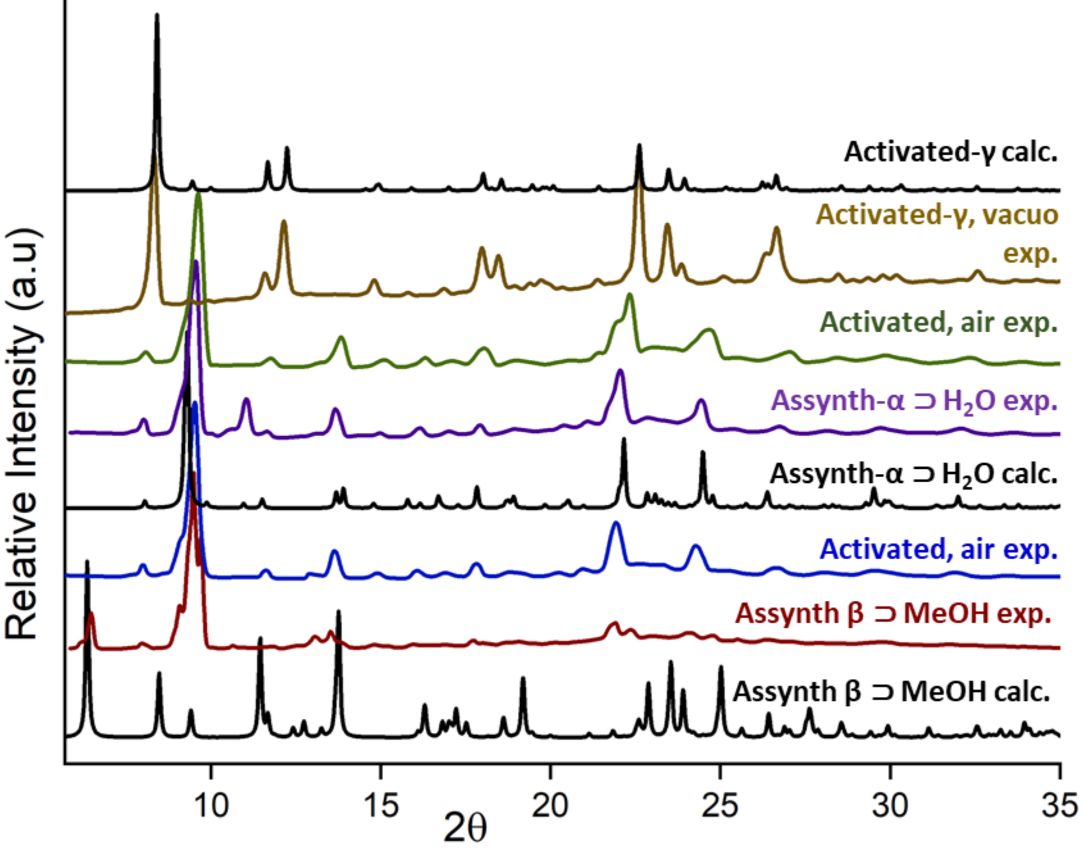

Figure S4. PXRD comparisons for calculated vs. experimental patterns for as-synthesised and activated phases.

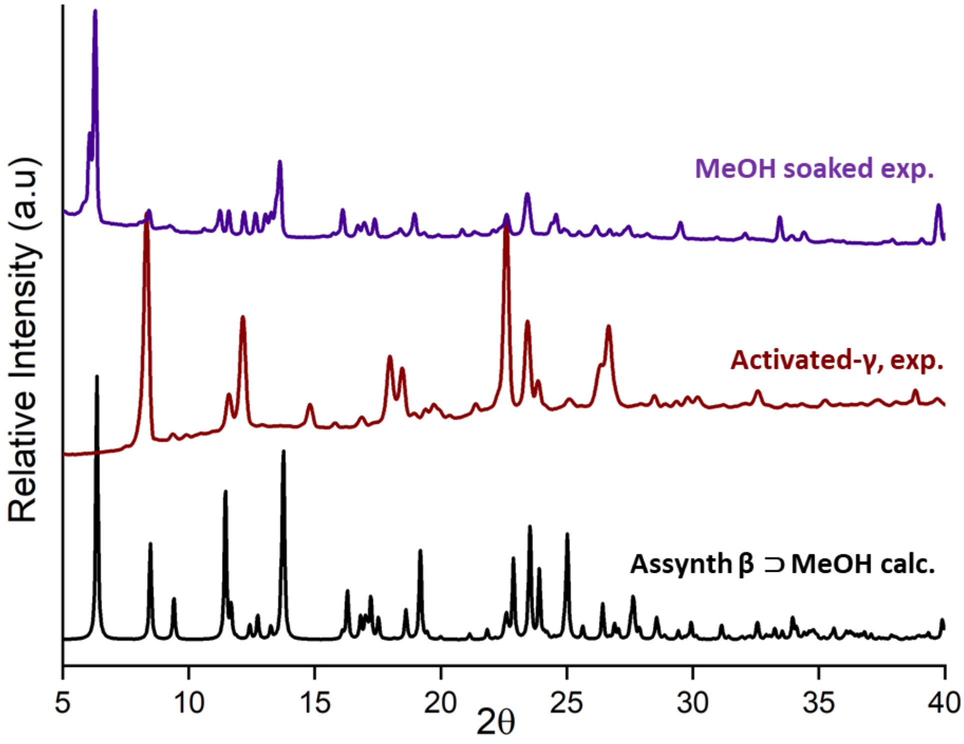

Figure S5. PXRD comparisons for as-synthesised β⊃MeOH (black), upon activation transform to γ⊃empty (red). Then γ⊃empty reverts to β⊃MeOH upon soaked in MeOH (purple).

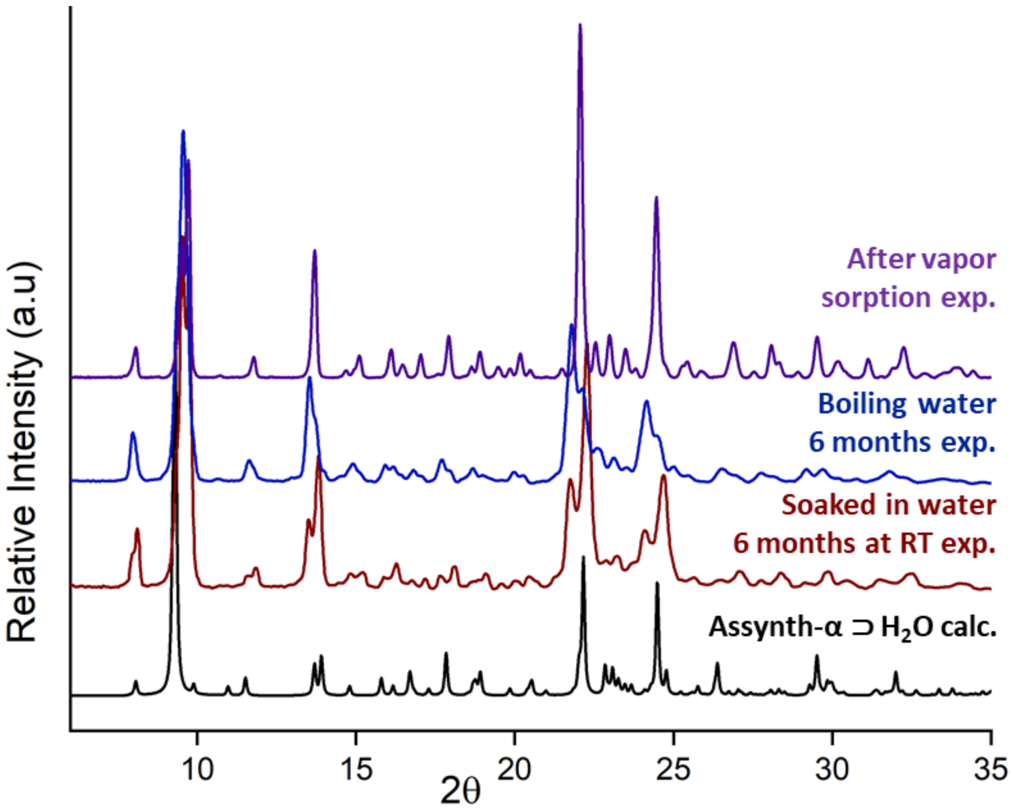

Figure S6. PXRD comparisons of sample obtained after vapor sorption experiments, water-soaked sample at room temperature or in boiling water for 6 months.

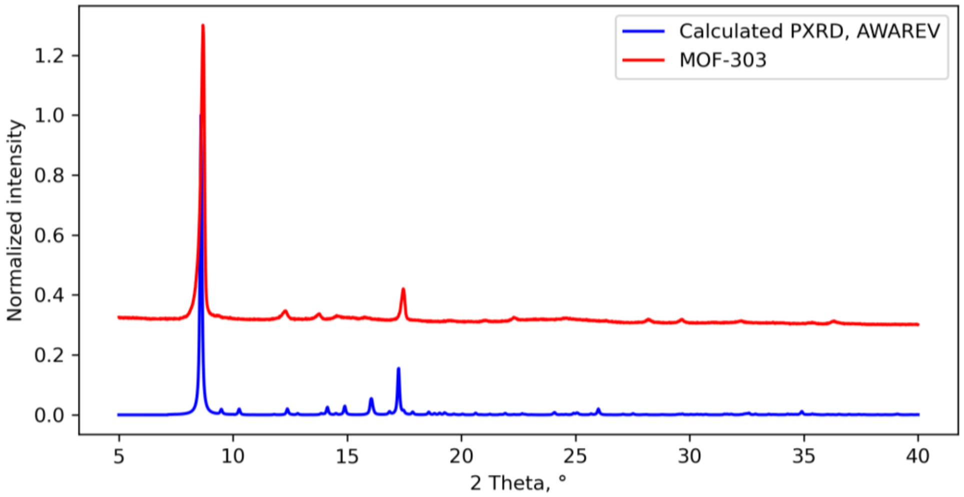

Figure S7. Comparison of experimental PXRD of as-synthesized MOF-303 powder and calculated pattern.[7]

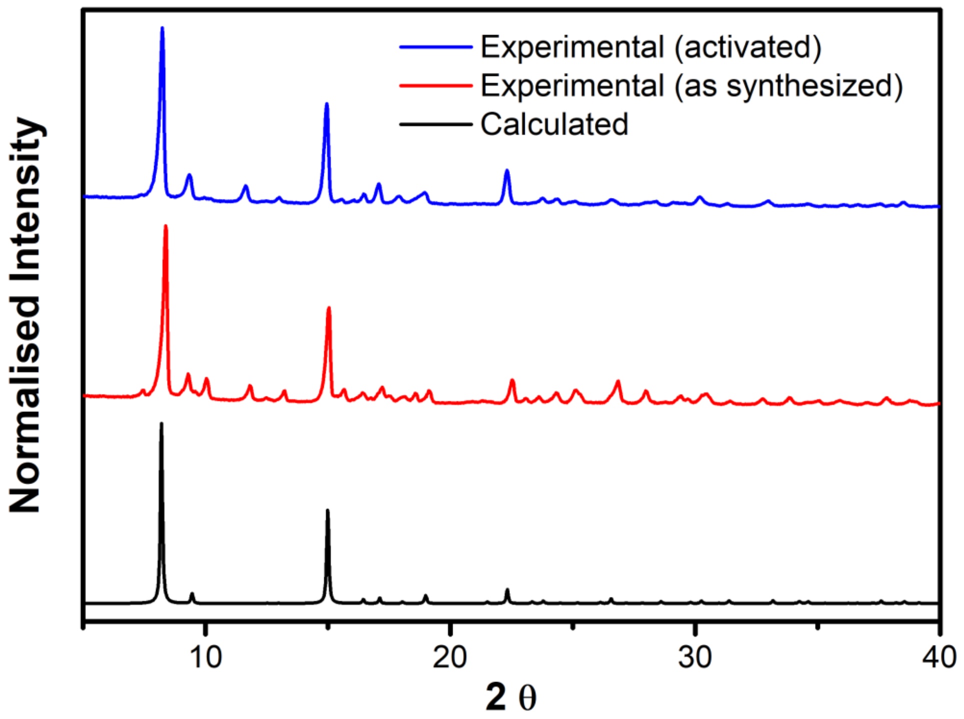

Figure S8. Comparison of experimental PXRD of as-synthesized CAU-10-H powder and calculated pattern.[2]

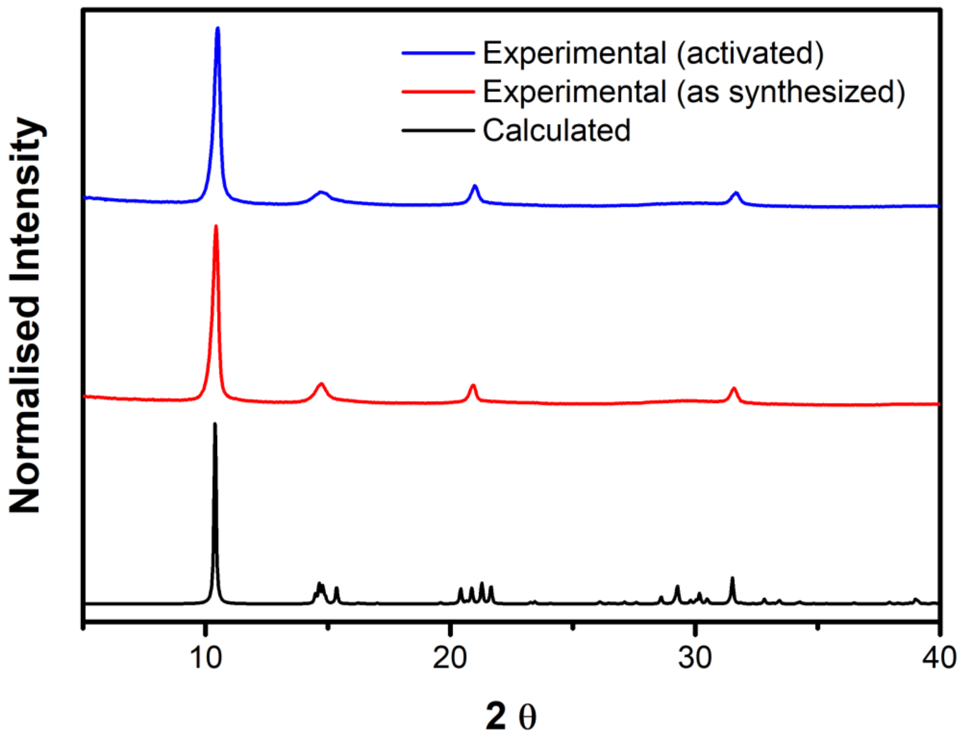

Figure S9. Comparison of experimental PXRD of as-synthesized Al-fumarate powder and calculated pattern.[8]

5. Thermogravimetric analysis

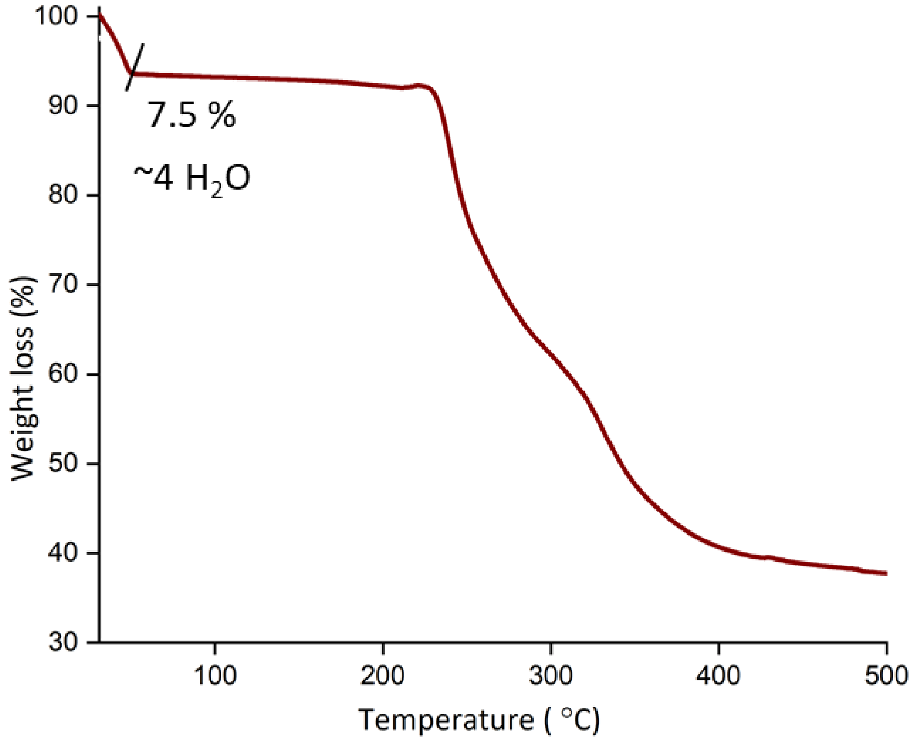

Figure S10. Thermogravimetric measurement for α⊃H_2O phase exhibits 7.5% weight loss that corresponds to 4 water molecules per formula unit. The final decomposition of the framework occurs at above ~230 °C.

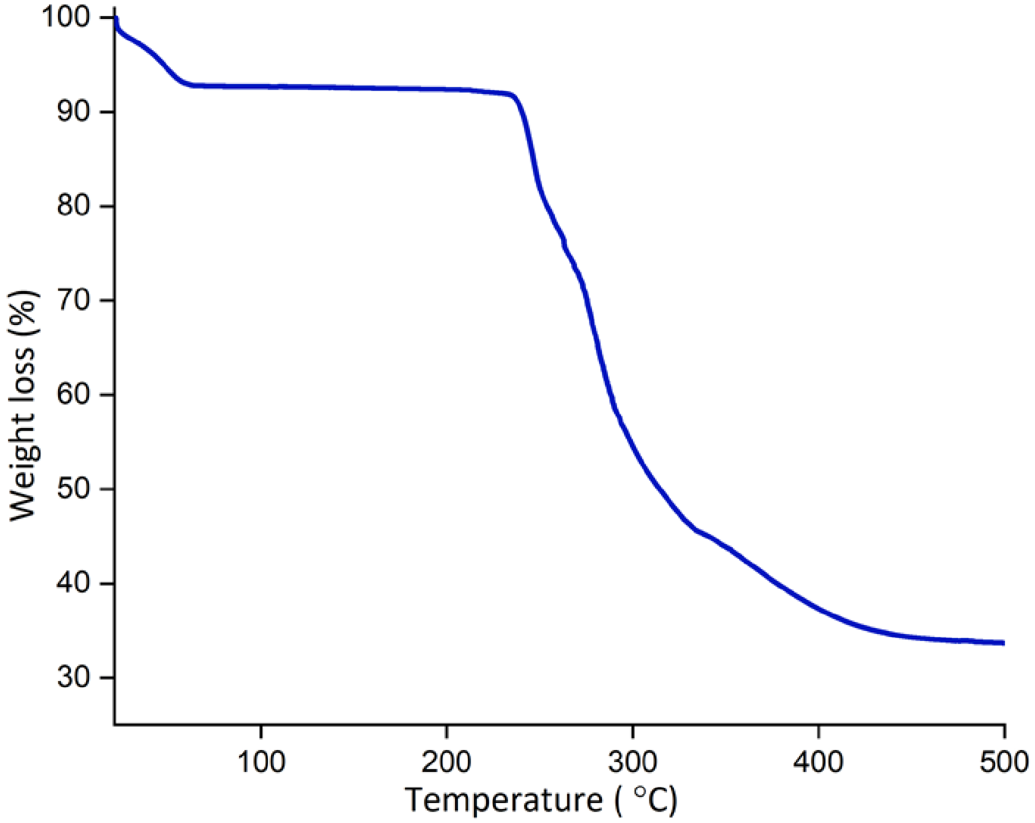

Figure S11. Thermogravimetric measurement for β⊃MeOH phase also shows 7.5% weight loss that corresponds to 3 methanol per formula unit. The framework is stable up to ~230 °C.

6. PXRD refinement

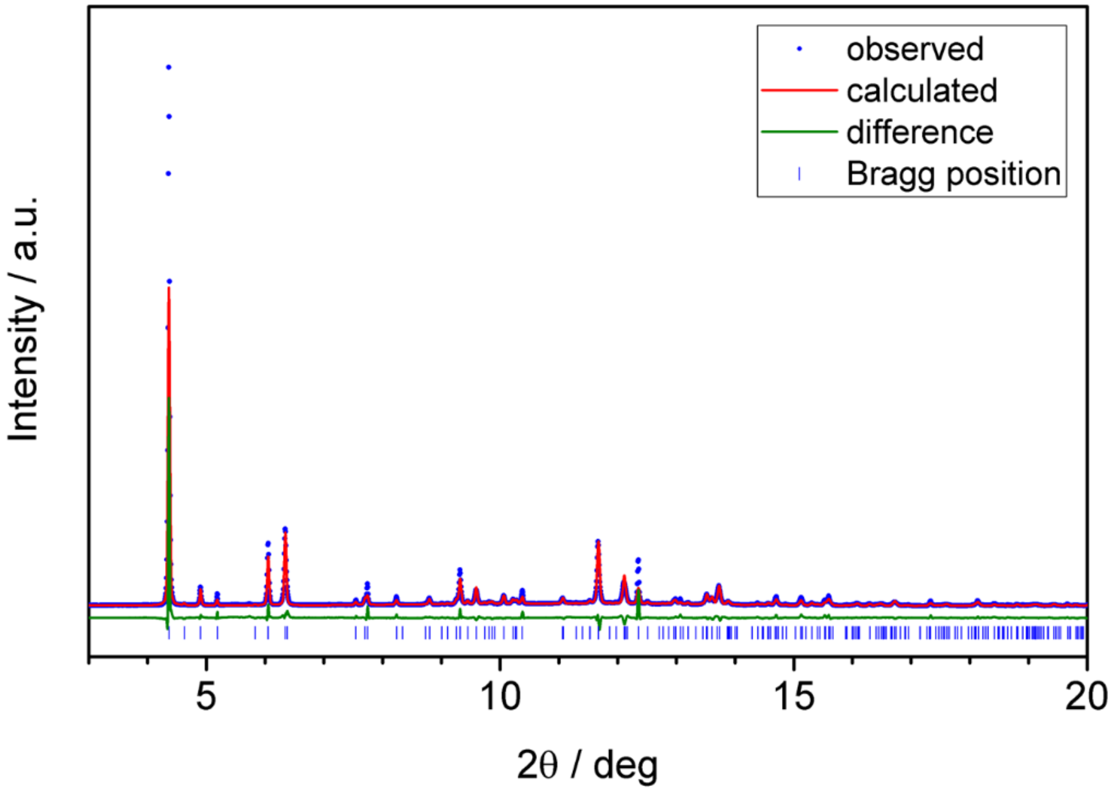

Figure S12. Rietveld refinement plot for γ phase structure determination.

7. Activated crystal structure and particle size measurements

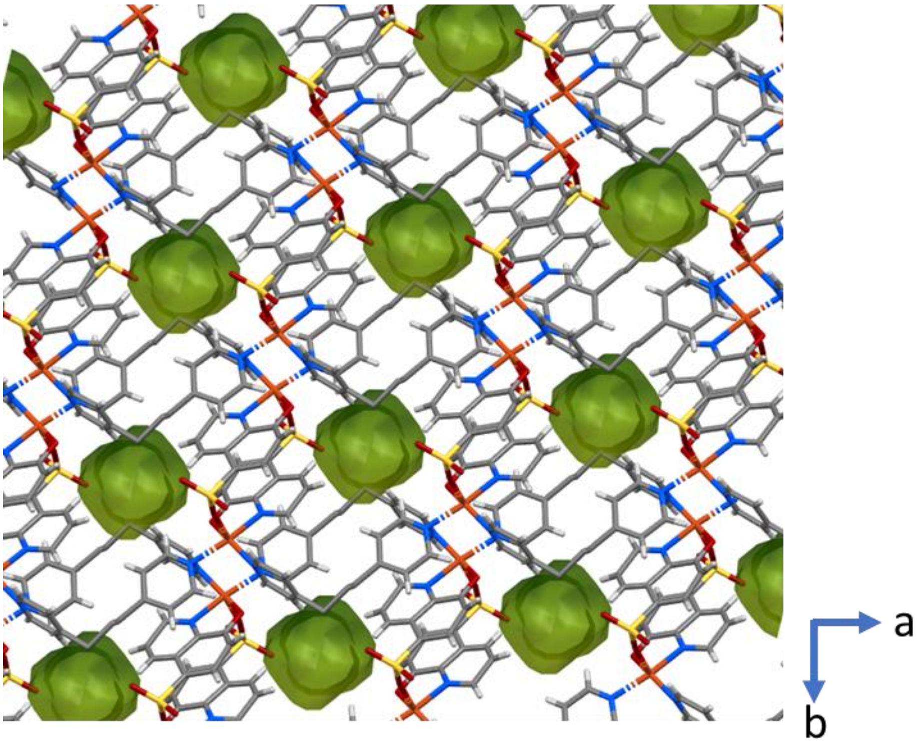

Figure S13. Closed packed structure of γ⊃empty phase and representation of 0D guest accessible channels (7.5%) calculated by considering probe radius of 1.2 Å and grid spacing of 0.7 Å.

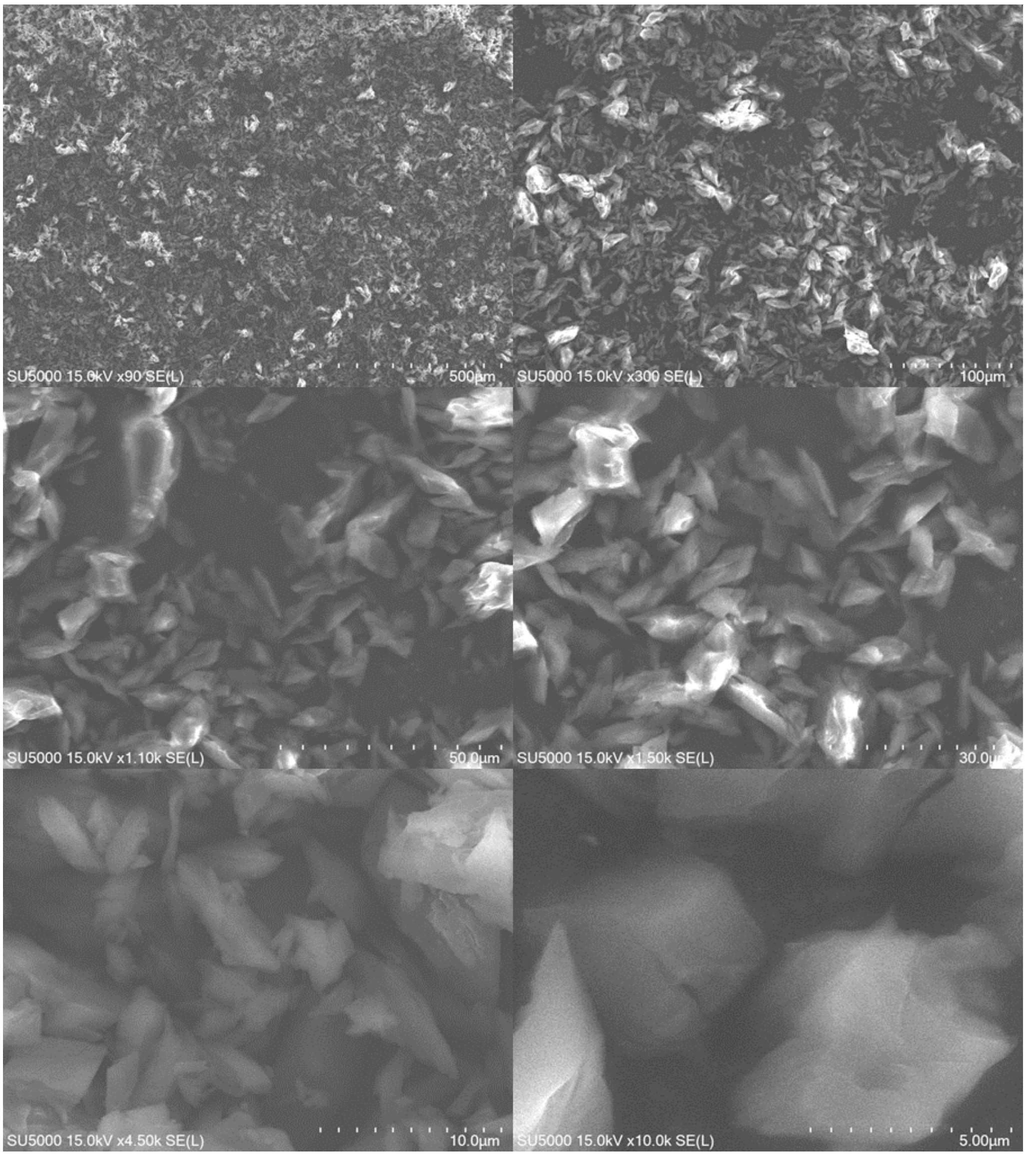

Figure S14. SEM images measured for the activated γ phase.

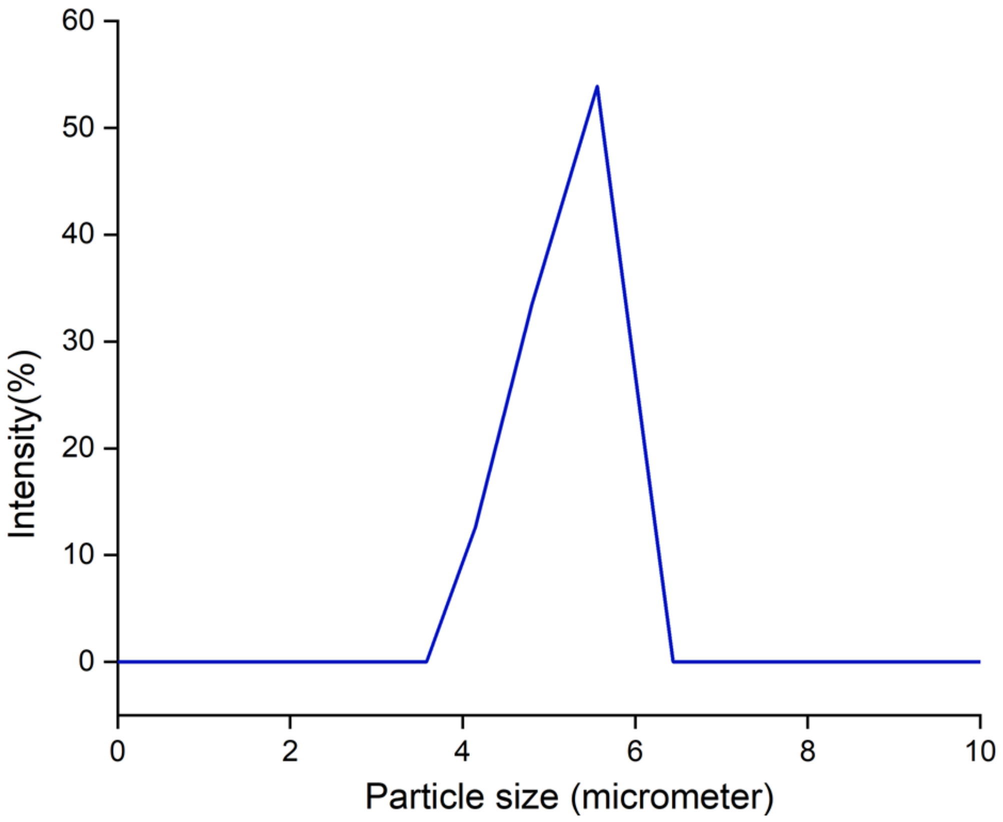

Figure S15. Particle size distribution measured for activated gamma sample dispersed in DMF.

8. Water vapor sorption isotherm and in-situ PXRD

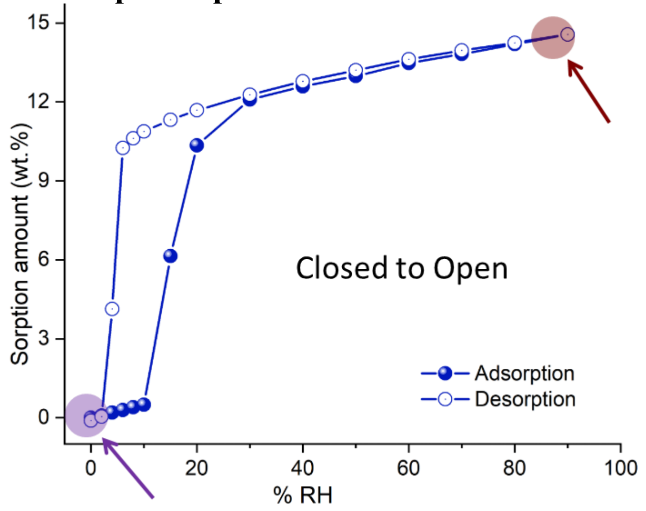

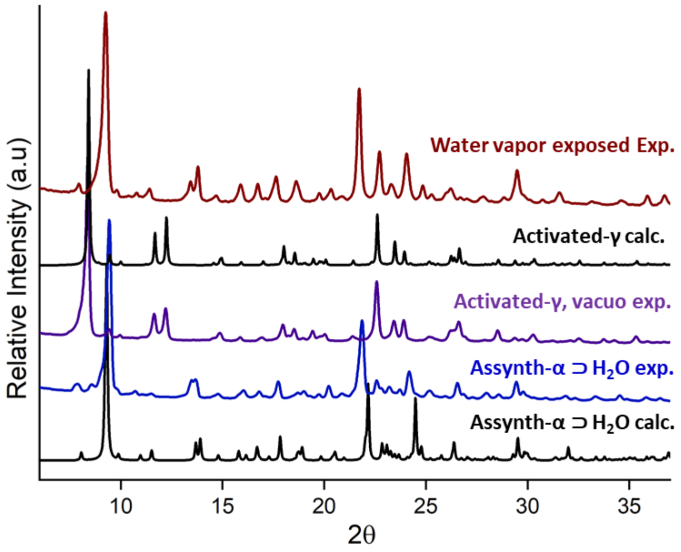

Figure S16. *In-situ* PXRD measurements for before (vacuo exp.) and after water loaded phases. The γ⊃empty phase transforms to α⊃H_2O when water vapour is exposed at room temperature and the phase purity of that batch compared with calculated pattern.

9. Reported porous sorbents for water vapor sorption

Table S2. Flexible sorbents for water vapor sorption and classifications based on their isotherm profile

No.	Sorbent name	Phase change	Isotherm type	References
1	(Fe)MIL-100	Open to more open, gradual/multiple	Type F-I	Adv. Mater. 2018, 30, 1704304
2	(Al)MIL-100	Open to more open, gradual/multiple	Type F-I	Adv. Mater. 2018, 30, 1704304
3	Cd(pzdc)(azpy)	Open to more open, gradual/multiple	Type F-I	Angew. Chem. Int. Ed. 2004, 43, 3269 –3272
4	Cd(pzdc)(bpee)	Open to more open, gradual/multiple	Type F-I	Angew. Chem. Int. Ed. 2004, 43, 3269 –3272
5	(Al)MIL-53(OH)	Open to more open, gradual/multiple	Type F-I	Adv. Mater. 2018, 30, 1704304
6	[Zn(oba)-(pip)]n (JUK-8)	Open to more open	Type F-I	Angew. Chem. Int. Ed. 2020, 59, 4491 – 4497
7	[Cu2(amp)4Cl][Cr(ox)3]	Open to more open, sudden	Type F-II	CrystEngComm, doi:10.1039/D2CE00138A (2022).
8	[Mn(imH)]2[Mo(CN)8]	Closed to open, gradual/multiple	Type F-III	Chem. Sci. 2021, 12, 9176-9188.
9	SIFSIX-23-Cu	Closed to open, gradual/multiple	Type F-III	J. Am. Chem. Soc. 2020, 142, 6896-6901.
10	(Cr)MIL-101 and its series	Closed to open, gradual/multiple	Type F-III	New J. Chem., 2014, 38, 3102—3111.
11	(Cr)MIL-100	Closed to open, gradual/multiple	Type F-III	Adv. Mater. 2018, 30, 1704304
12	[Zn(H2SSA)2-(H_2O)2]7.1H_2O	Closed to open, through multiple	Type F-III	Chem. Commun., 2019, 55, 9713–-9716
13	DMOF-TM2 (unstable in liquid water)	Closed to open, sudden single step	Type F-IV	Langmuir 2013, 29, 633−642.
14	(Cr)MIL-53 (in complete desorption and large hysteresis)	large to narrow pore, sudden single step	Type F-IV	New J. Chem., 2014, 38, 3102—3111. J. Am. Chem. Soc. 2010, 132, 9488-9498.
15	DUT-98 and its series (particle size dependent)	Closed to open, sudden single step	Type F-IV	Beilstein J. Nanotechnol. 2019, 10, 1737–1744
16	This work	Closed to open, sudden single step	Type F-IV	

10. Water vapor sorption on different phases and at different temperatures

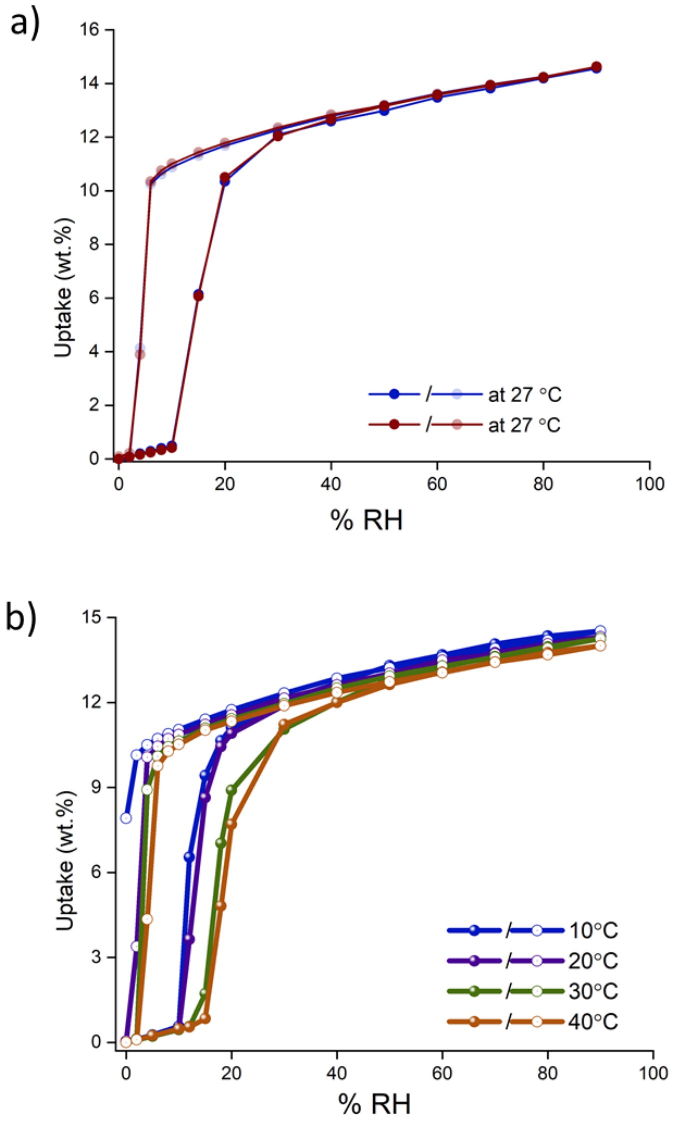

Figure S17. a) Water vapor sorption isotherms measured for γ phase activated from α (blue) compared with the γ phase activated from β (red). b) Isotherms at different temperatures (10, 20, 30, 40 °C) measured using Intrinsic-DVS instrument for the γ phase obtained from the β.

11. Water vapor sorption for reported sorbents

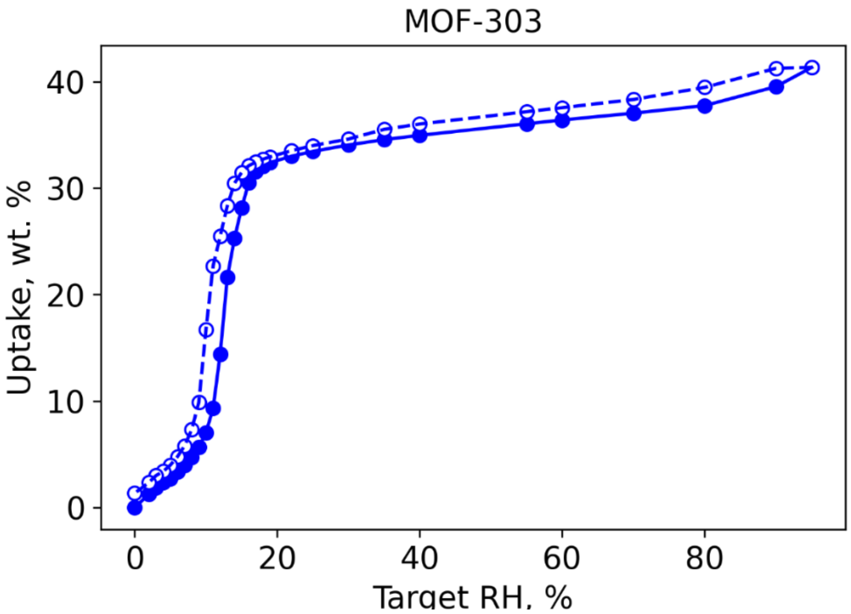

Figure S18. Water vapor sorption isotherm of MOF-303 measured at 27 °C using Adventure-DVS instrument.

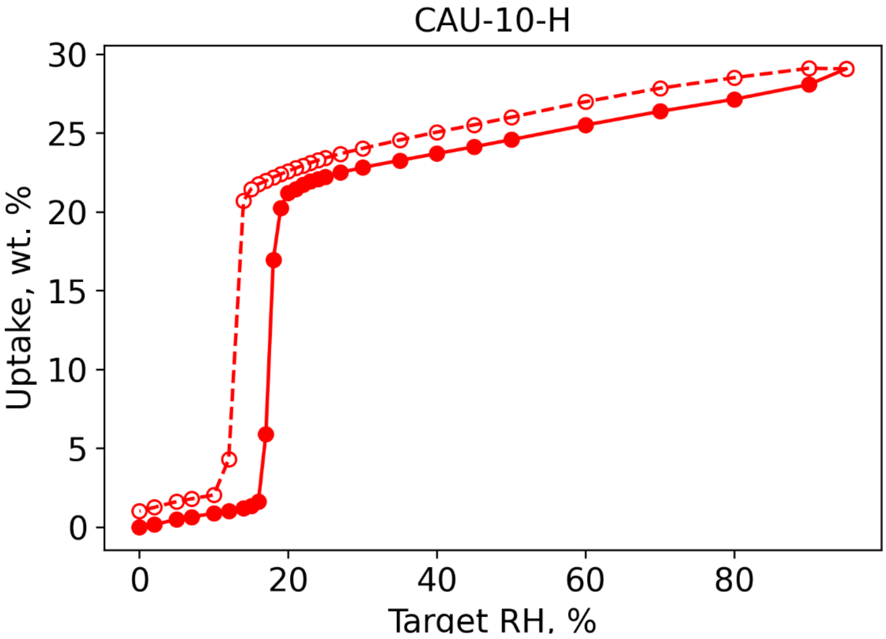

Figure S19. Water vapor sorption isotherm of CAU-10-H measured at 27 °C using Adventure-DVS instrument.

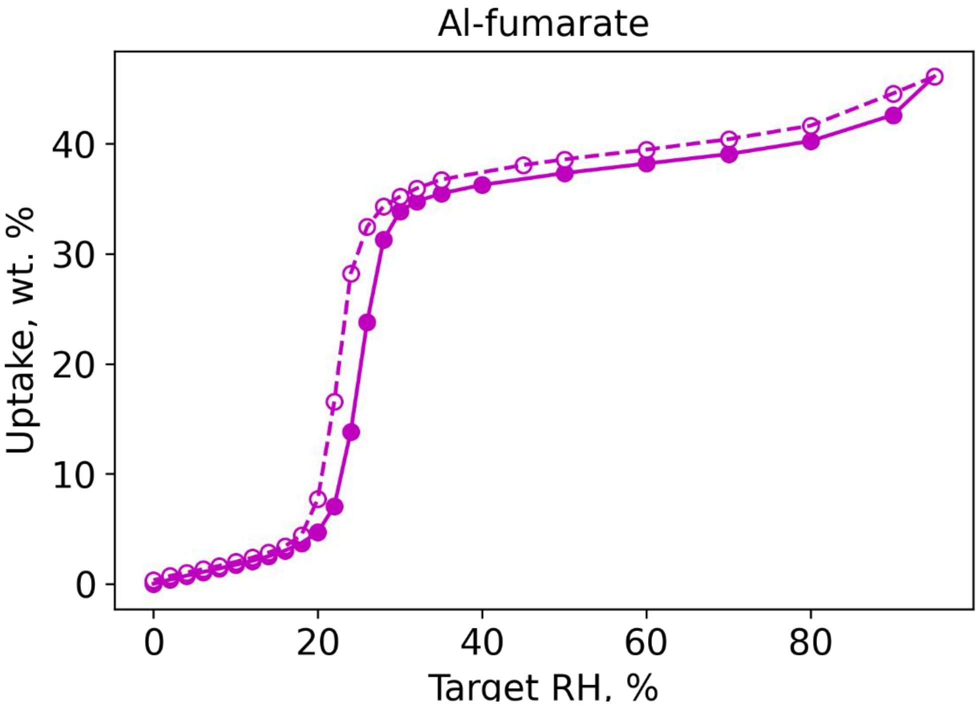

Figure S20. Water vapor sorption isotherm of Al-fumarate measured at 27 °C using Adventure-DVS instrument.

12. Kinetics and water sorption cycles

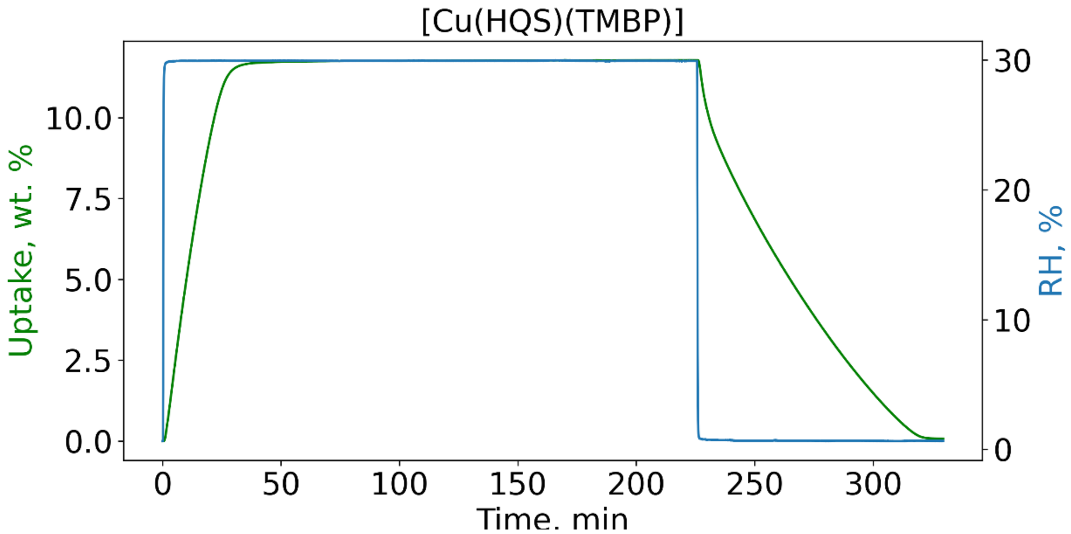

Figure S21. Water vapor sorption kinetics on 9.28 mg sample of [Cu(HQS)(TMBP)] measured at 27 °C using Adventure-DVS instrument.

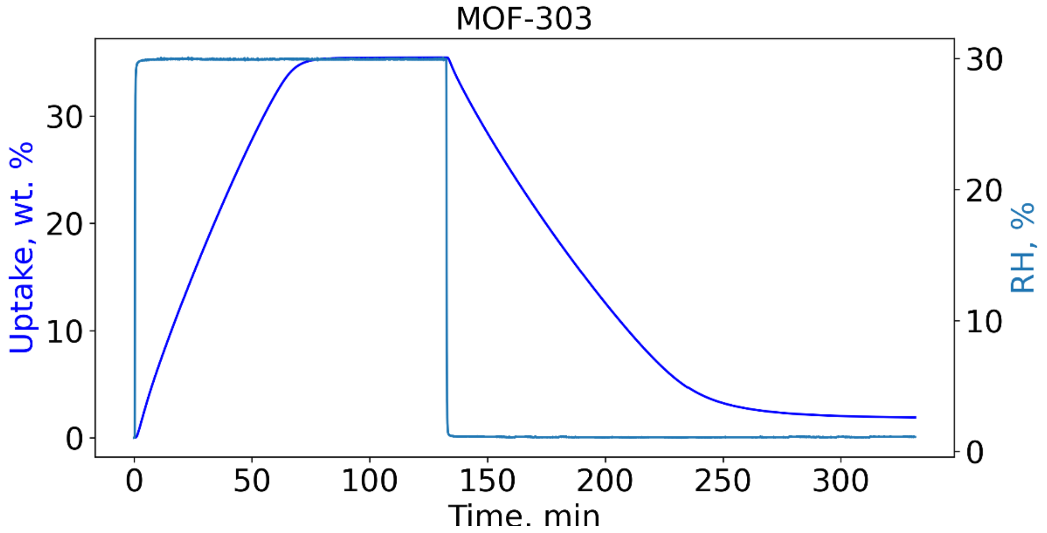

Figure S22. Water vapor sorption kinetics on 11.29 mg sample of MOF-303 measured at 27 °C using Adventure-DVS instrument.

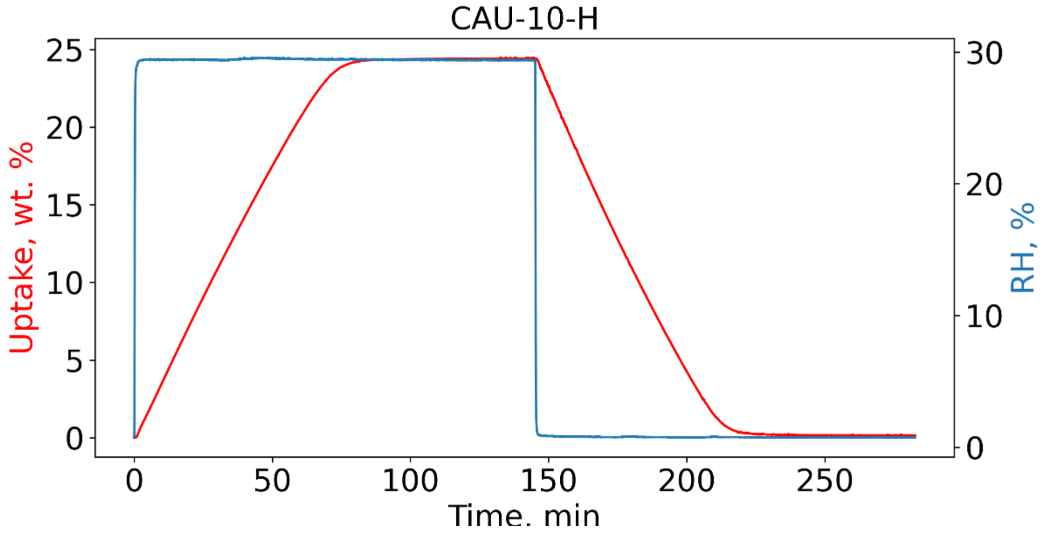

Figure S23. Water vapor sorption kinetics on 11.34 mg sample of CAU-10-H measured at 27 °C using Adventure-DVS instrument.

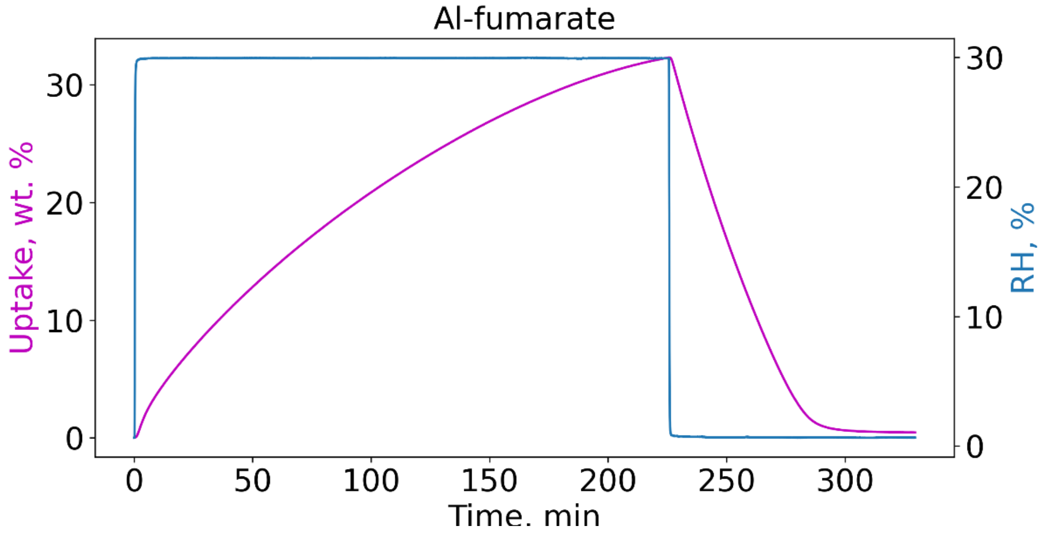

Figure S24. Water vapor sorption kinetics on 12.05 mg sample of Al-fumarate measured at 27 °C using Adventure-DVS instrument.

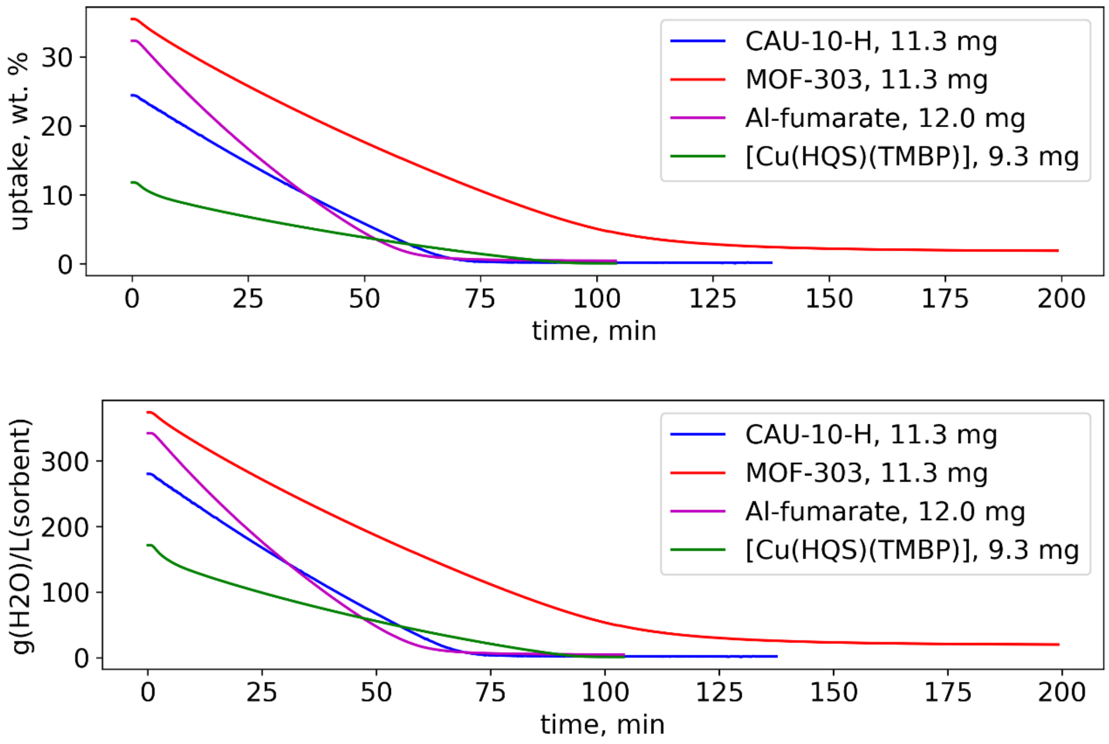

Figure S25. Comparison of the kinetics of water unloading on [Cu(HQS)(TMBP)] with three leading rigid MOF sorbents, MOF-303, CAU-10-H and Al-fumarate in (300 K, 30 % RH) to (300 K, 0 % RH) when subjected to humidity swing cycling.

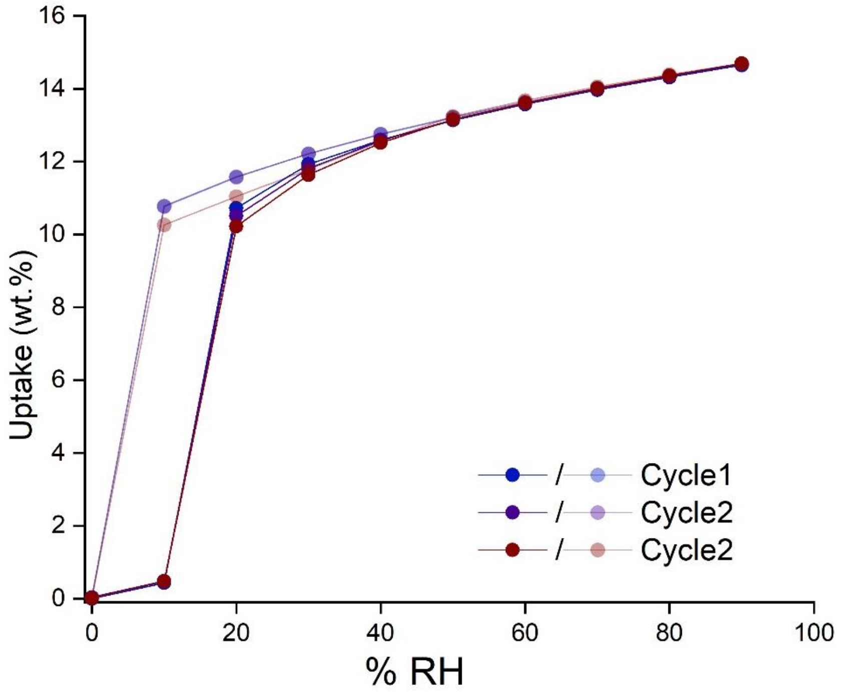

Figure S26. Three consecutive water vapor sorption isotherms measured at 27 °C using Intrinsic-DVS instrument.

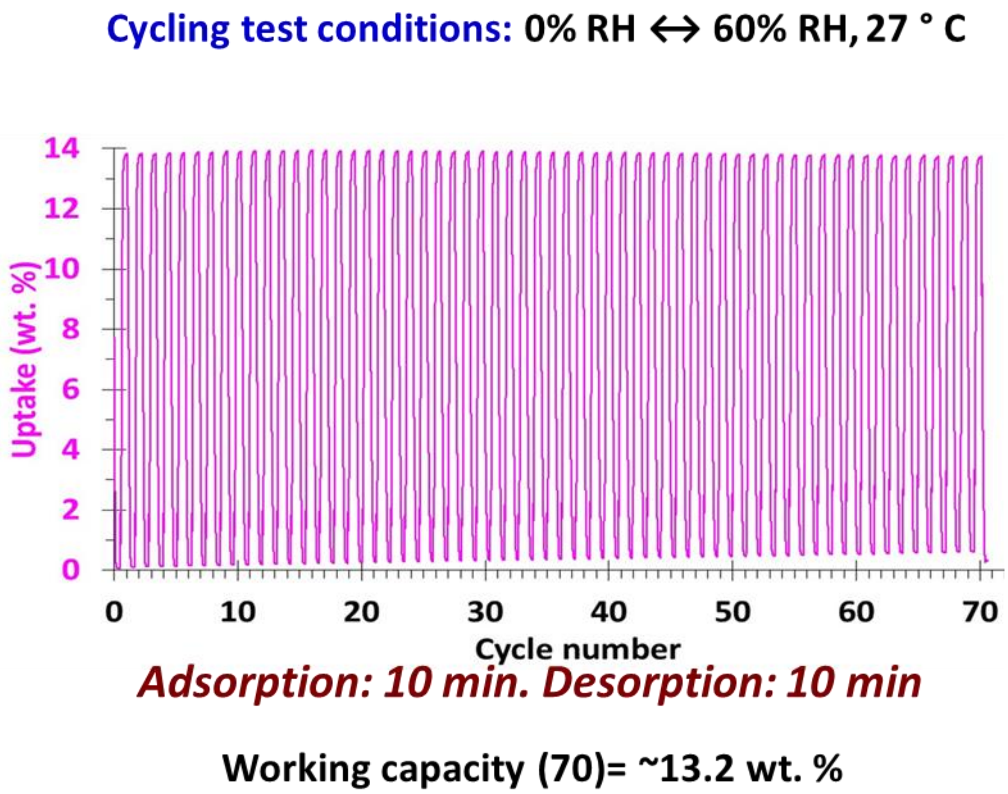

Figure S27. 70 water vapor cycling experiments were measured on coordination network material. This experiment was performed in the Vacuum-DVS instrument. In every cycle the material was loaded at 27 °C from 0% RH to 60% RH for 10 min followed by desorption at 27 °C for 10 min from 60% RH to 0% RH. The mass at the end of the first 0% RH stage was 10.1752 mg. The deliverable working capacity calculated as the difference between the uptake under adsorption and desorption conditions at the end of the last cycle was 13.2 wt. %. Before starting the cycling tests, the sample was in-situ pre-activated at 0% RH and 50 °C for 2 h and at 27 °C for 1 h under high vacuum pressure (turbo pump on). After these cycling experiments or further continuous cycling tests, we found that powder sample converts to foam but after heat treatment, foam again reverts to powder shape. Interestingly, the PXRD for the powder which is obtained from the foam is match with the alpha.

13. Composite preparation, characterization and water vapor sorption

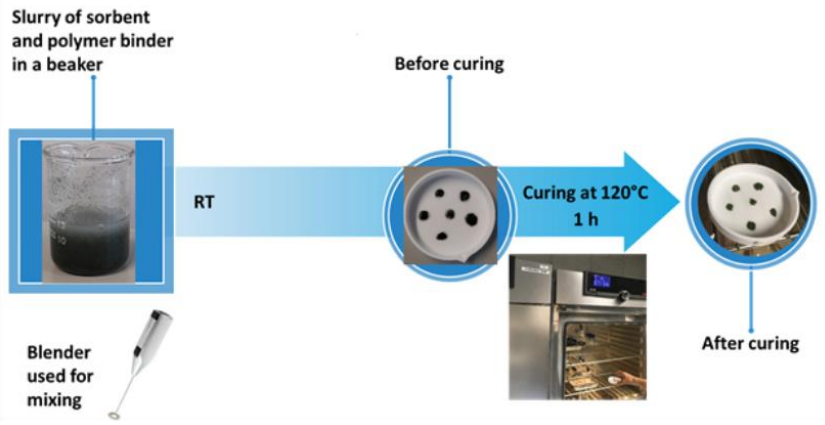

Binder: Acrylic Polymer: HYCAR® 26410 (Lubrizol)

Physical and chemical properties: pH = 4.4 – 5.2; Boiling point = 100 °C; Freezing point: ~ 0 °C
Relative density = 1.05 – 1.09 (@20 °C)

Composite	Coordination network (g)	Binder (g)	Isopropanol (g)	Water (g)
Coordination network:polymer	0.55	0.5	3.85	9.7
Pure polymer	0	0.5	3.85	9.7

Figure S28. Schematic representation of synthesis of coordination network-polymer composite and corresponding pure polymer. Supplied details for polymer used and their concentrations.

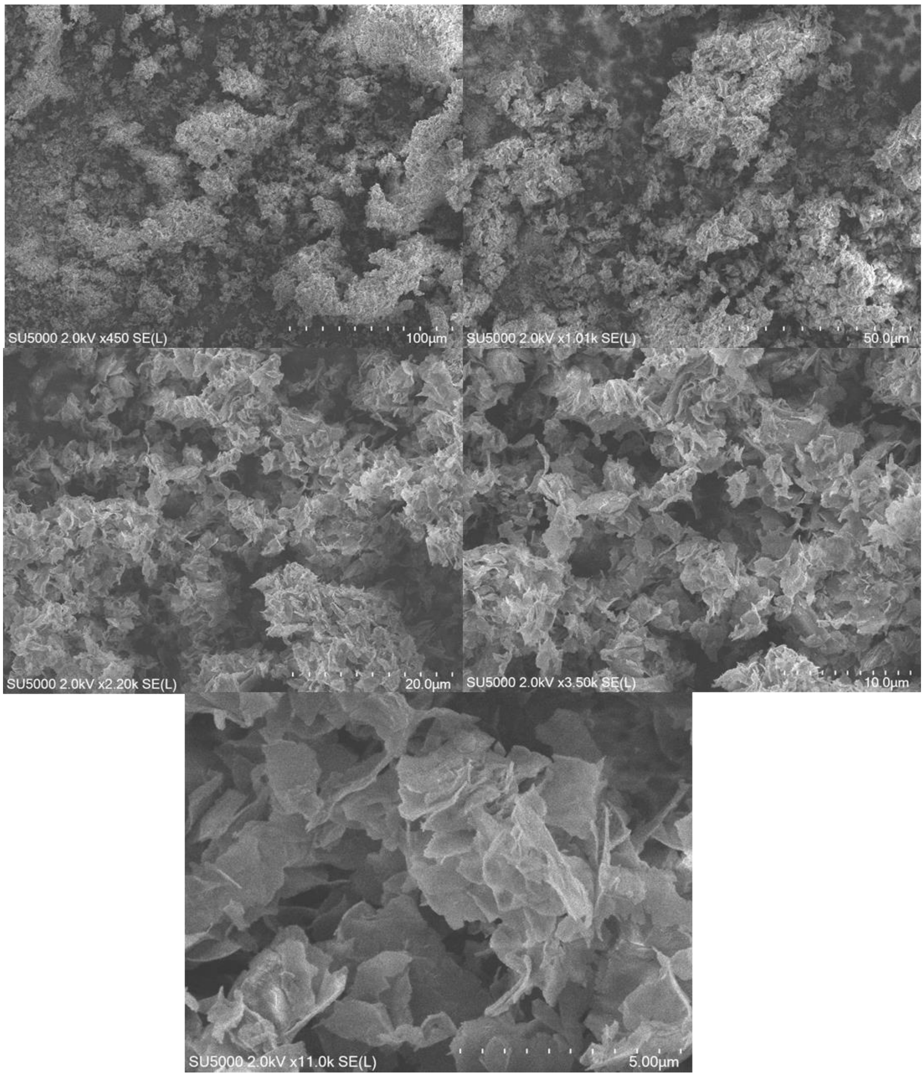

Figure S29. SEM images were measured for the composite. Indicated that homogenous mixture of MOF and polymer.

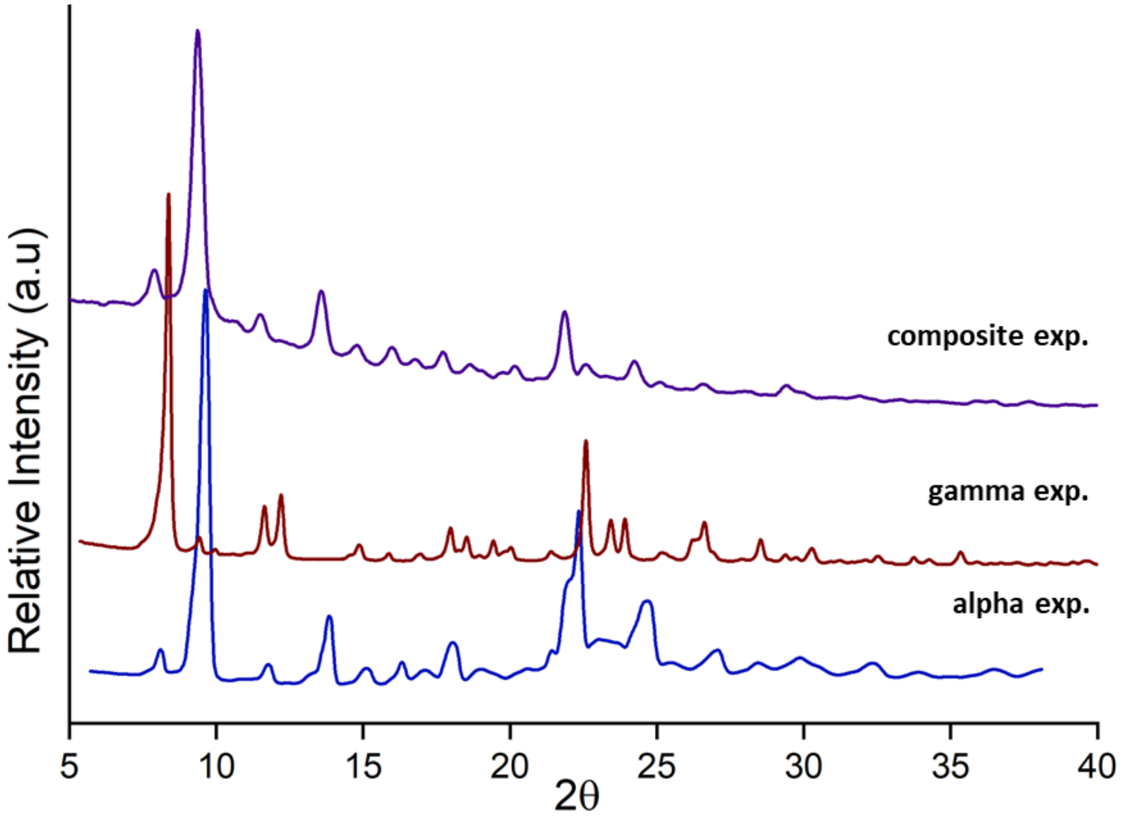

Figure S30. PXRD comparisons for as-synthesised α⊃H_2O (blue), γ⊃empty (red) and coordination network-polymer composite (purple).

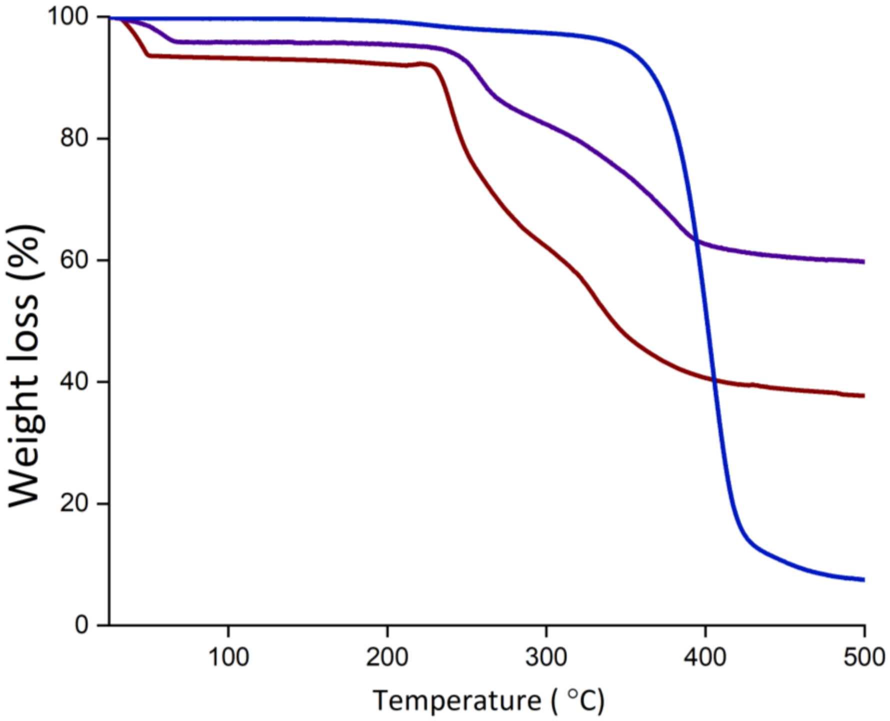

Figure S31. Thermogravimetric measurement of α⊃H_2O phase (red), coordination network-polymer composite (purple) and pure polymer (blue).

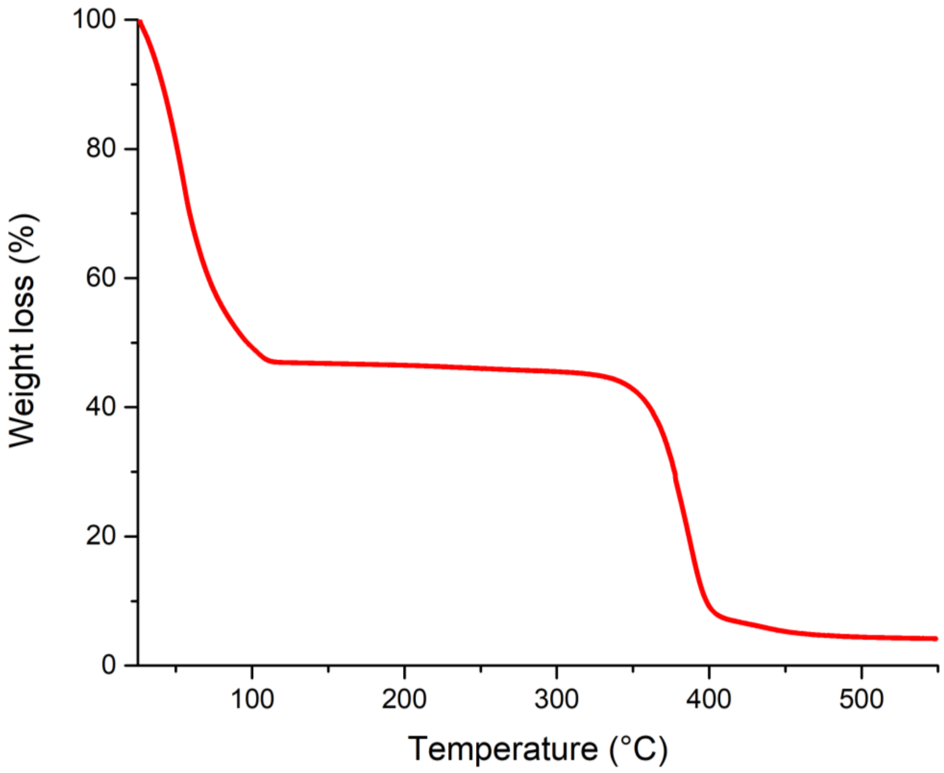

Figure S32. Thermogravimetric measurement of uncured HYCAR® 26410 polymer binder.

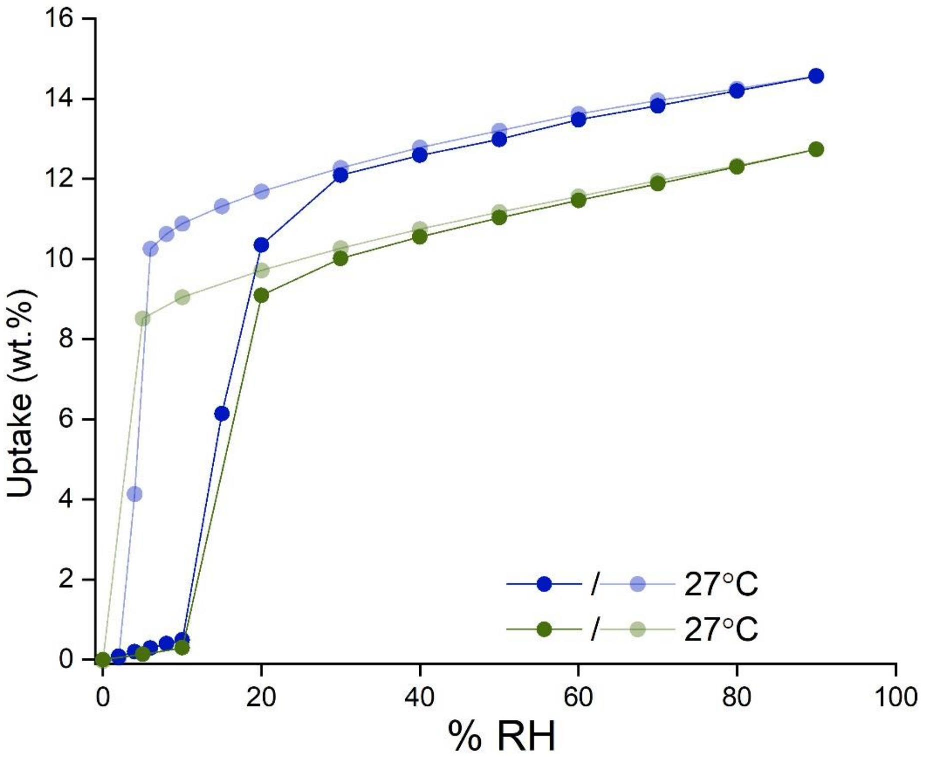

Figure S33. Water vapor sorption isotherms measured for pure coordination network (blue) and [Cu(HQS)(TMBP)]-polymer composite at 27 °C (green). These experiments were performed in the Intrinsic-DVS instrument.

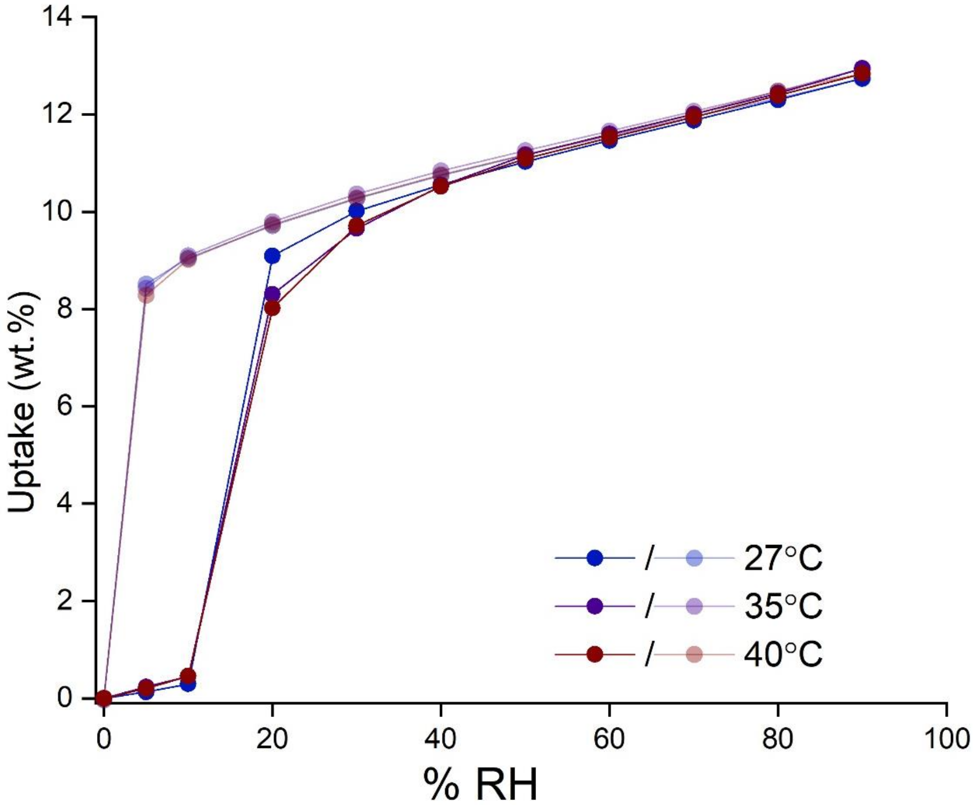

Figure S34. Water vapor sorption isotherms measured for [Cu(HQS)(TMBP)]-polymer composite at different temperatures, 27, 35 and 40 °C. These experiments were performed in the Intrinsic-DVS instrument.

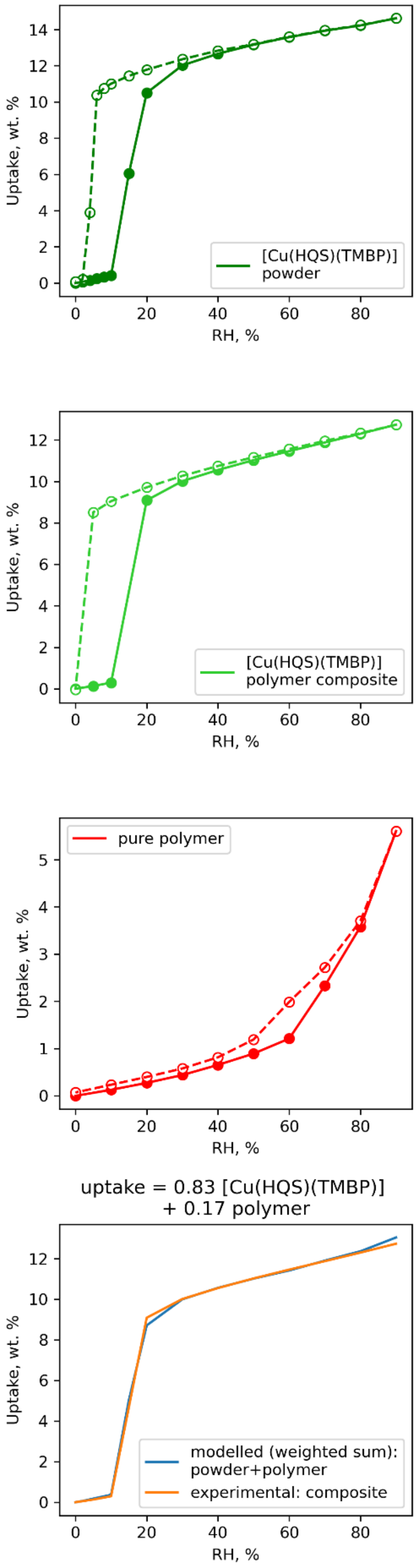

Figure S35. Top to bottom: water vapour sorption isotherms of pure [Cu(HQS)(TMBP)] powder, pure polymer, [Cu(HQS)(TMBP)]-polymer composite, comparison of weighted sum of [Cu(HQS)(TMBP)] powder and pure polymer adsorption isotherms with experimental [Cu(HQS)(TMBP)]-polymer composite adsorption isotherm.

14. Water vapor sorption cycling on composite

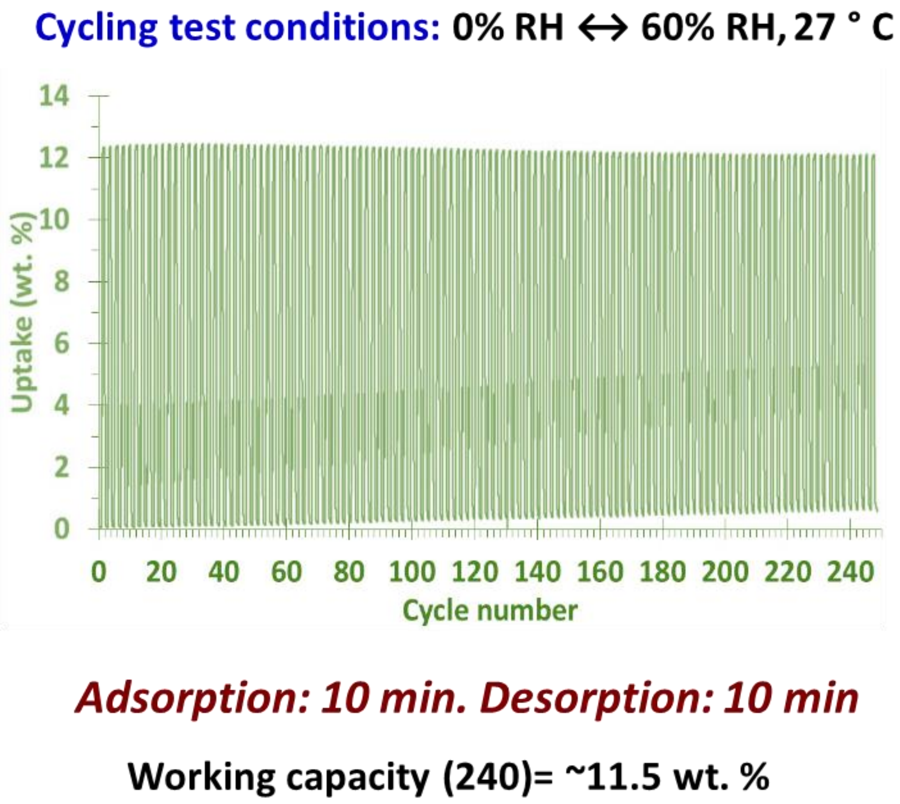

Figure S36. 248 water vapor cycling experiments were measured on coordination network-polymer composite material. This experiment was performed in the Vacuum-DVS instrument. In every cycle the material was loaded at 27 °C from 0% RH to 60% RH for 10 min followed by desorption at 27 °C for 10 min from 60% RH to 0% RH. The mass at the end of the first 0% RH stage was 4.4738 mg. The deliverable working capacity calculated as the difference between the uptake under adsorption and desorption conditions at the end of the last cycle was 11.5 wt. %. Before starting the cycling tests, the sample was in-situ pre-activated at 0% RH and 50 °C for 2 h and at 27 °C for 1 h under high vacuum pressure (turbo pump on).

15. Crystal structure analysis

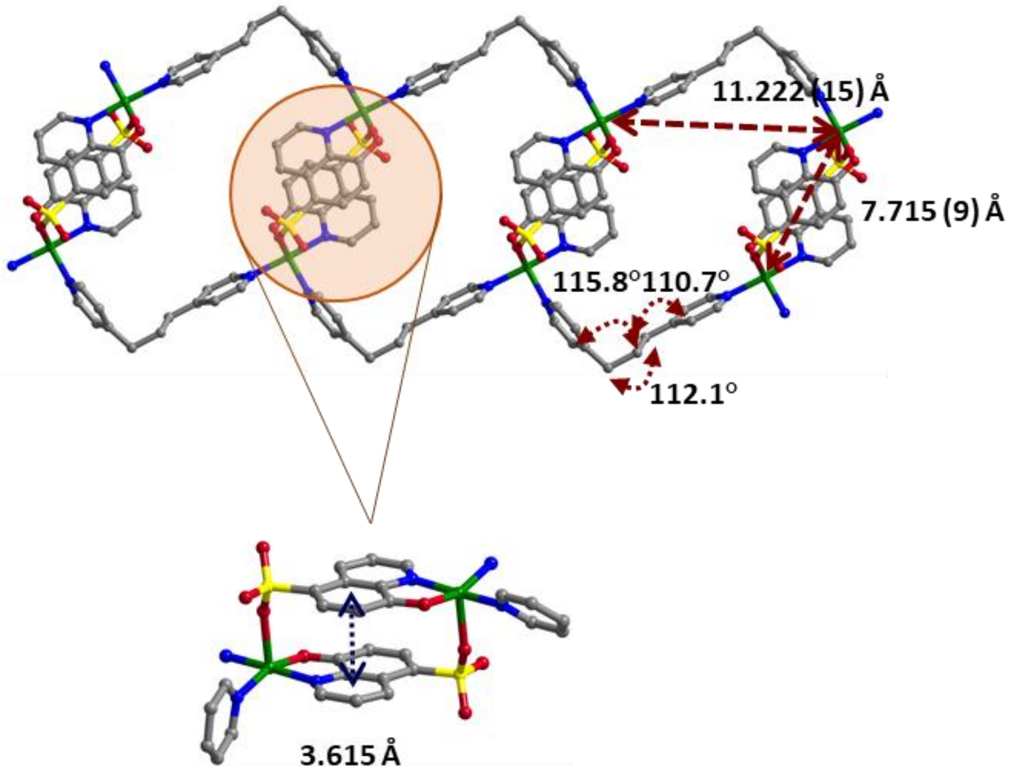

Figure S37. Representation of distances, π···π contacts in HQS, orientation angle of alkane chain in TMBP for α⊃H_2O structure.

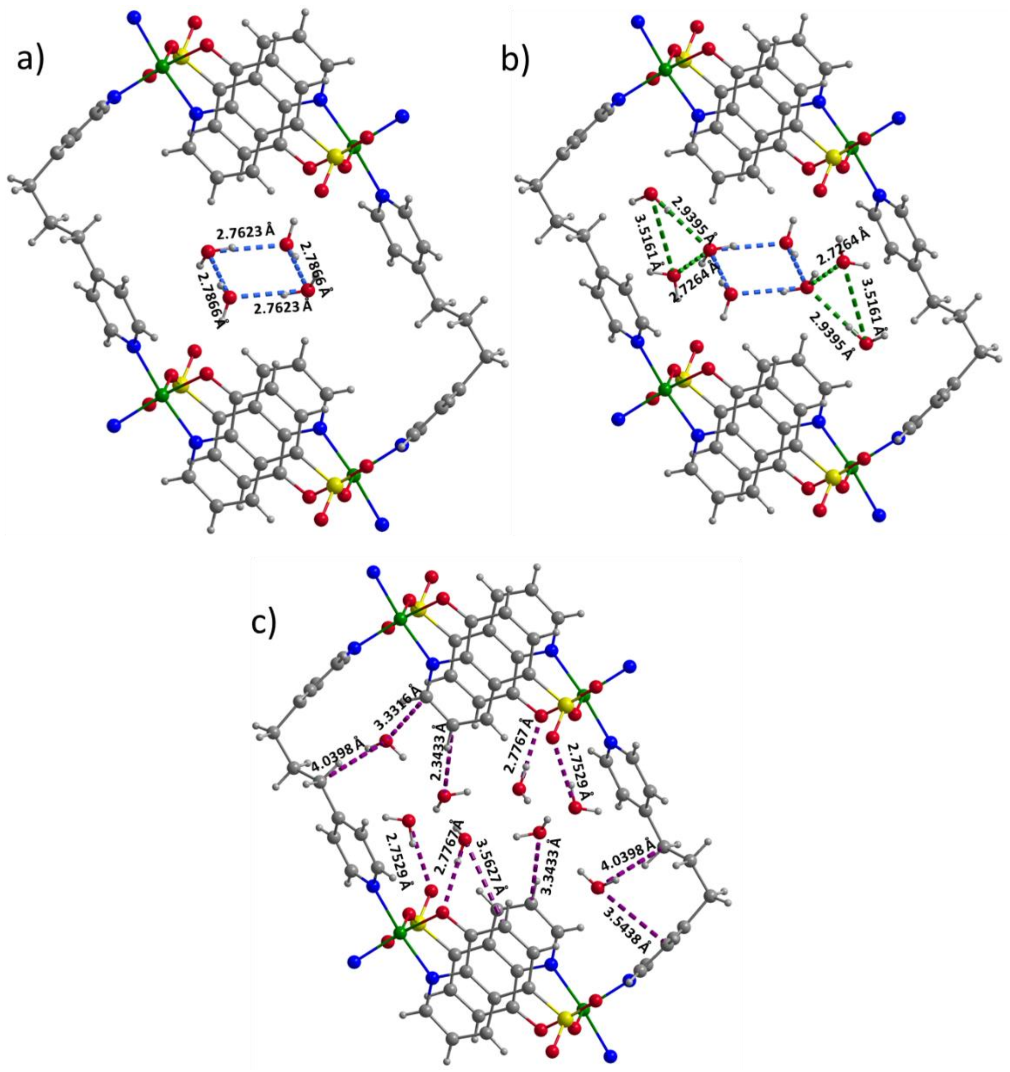

Figure S38. Representation hydrogen bonding interactions of square water clusters (a), then it is H-bonded to four water molecules in the form of octameric cluster (b), and multiple interactions between water and functional groups of host framework in α⊃H_2O phase (c).

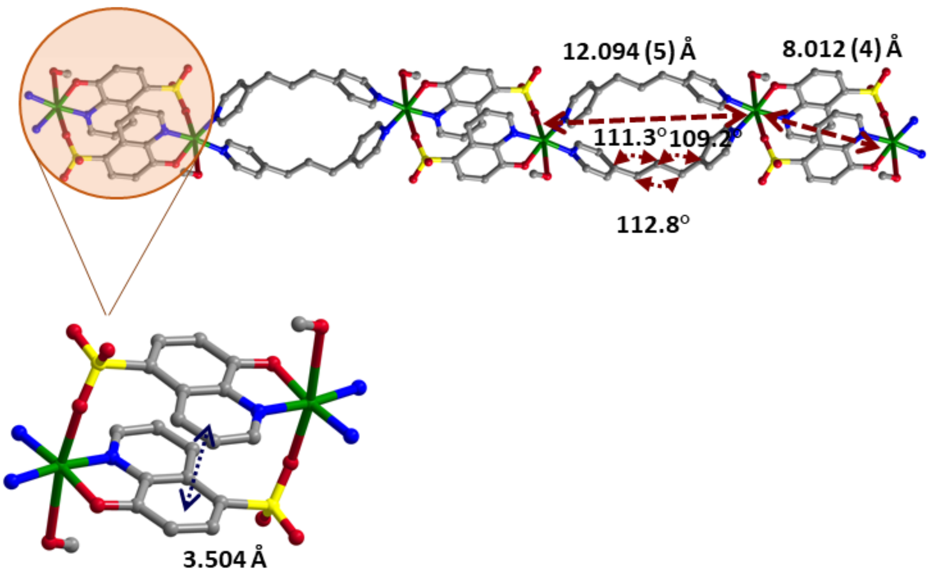

Figure S39. Representation of distances, π···π contacts in HQS, orientation angle of alkane chain in TMBP for β⊃MeOH structure.

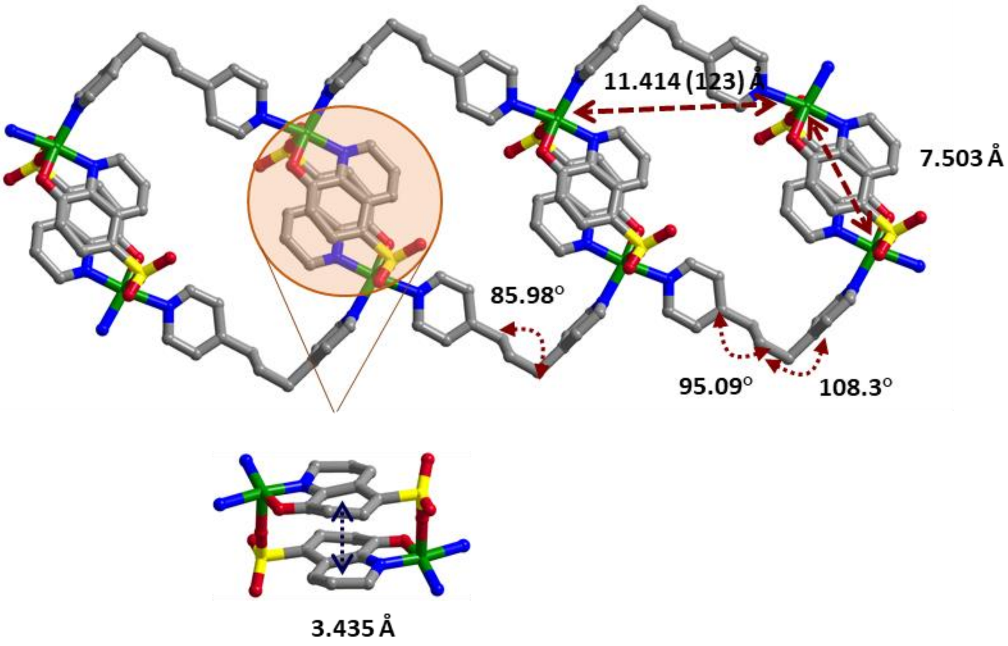

Figure S40. Representation of distances, π···π contacts in HQS, orientation angle of alkane chain in TMBP for γ⊃empty structure.

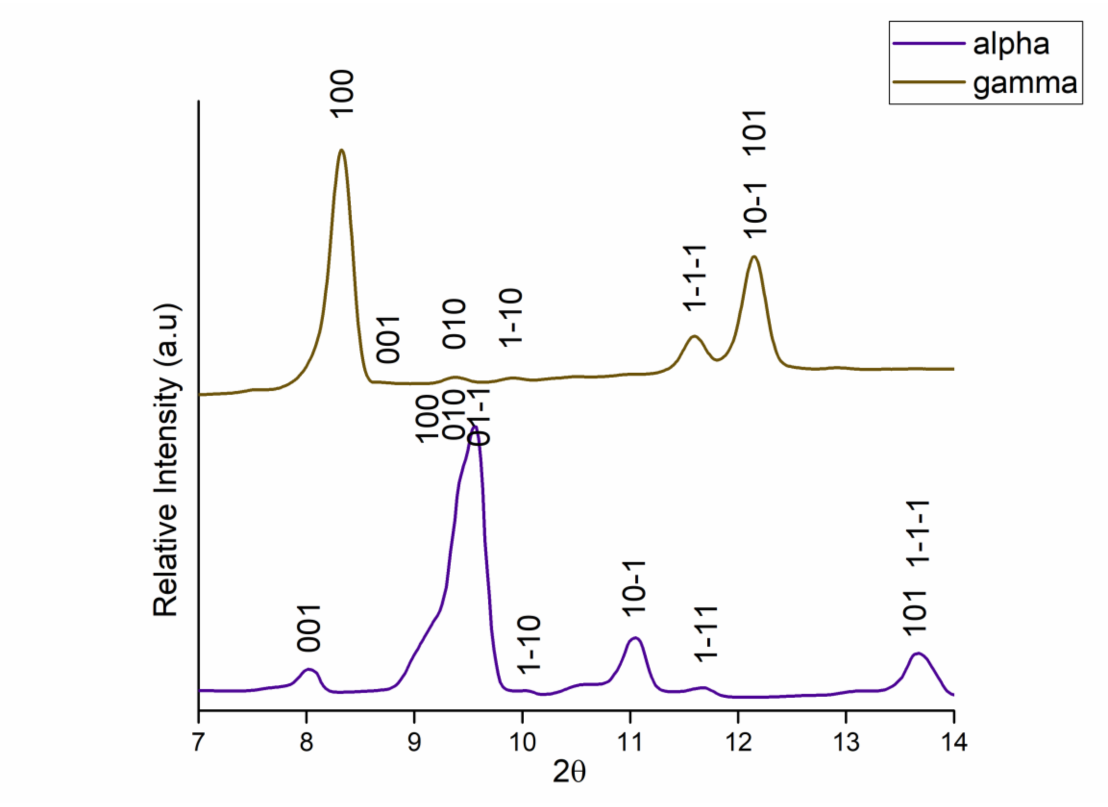

Figure S41. Experimental powder X-ray diffraction patterns of alpha and gamma phases with labeled reflections.

a)

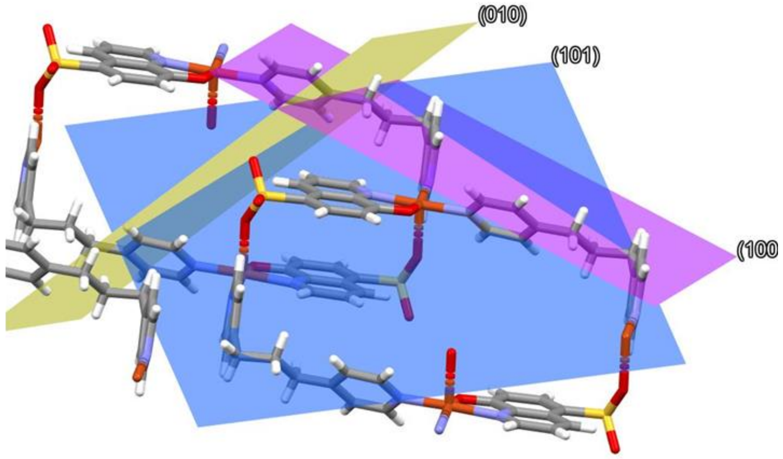

b)

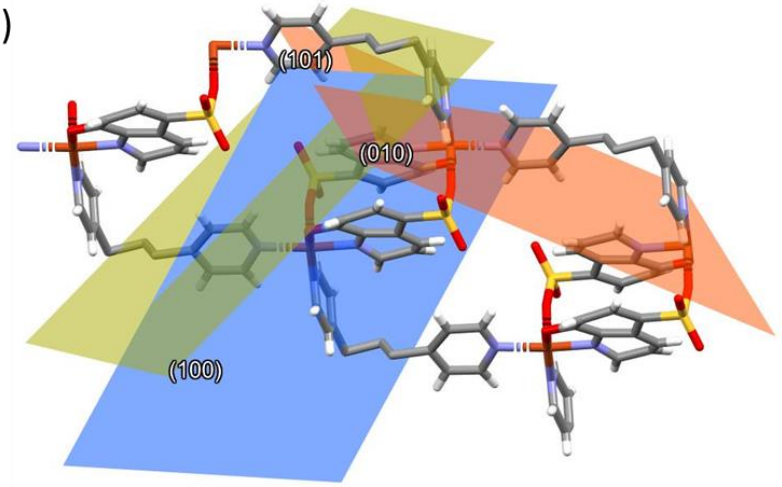

Figure S42. Representation of few significant refection planes in crystal structures of alpha (a) and gamma (b) phases.

16. References

[1] F. Jeremias, D. Fröhlich, C. Janiak, S. K. Henninger, *RSC Advances* **2014**, *4*, 24073-24082.

[2] H. Reinsch, M. A. van der Veen, B. Gil, B. Marszalek, T. Verbiest, D. de Vos, N. Stock, *Chemistry of Materials* **2013**, *25*, 17-26.

[3] N. Hanikel, M. S. Prevot, F. Fathieh, E. A. Kapustin, H. Lyu, H. Wang, N. J. Diercks, T. G. Glover, O. M. Yaghi, *ACS Cent Sci* **2019**, *5*, 1699-1706.

[4] W. I. F. David, K. Shankland, J. van de Streek, E. Pidcock, W. D. S. Motherwell, J. C. Cole, *Journal of Applied Crystallography* **2006**, *39*, 910-915.

[5] B. H. Toby, R. B. Von Dreele, *Journal of Applied Crystallography* **2013**, *46*, 544-549.

[6] aG. M. Sheldrick, *Acta Cryst.* **2015**, *A71*, 3-8; bG. M. Sheldrick, *Acta Cryst.* **2015**, *C71*, 3-8.

[7] F. Fathieh, M. J. Kalmutzki, E. A. Kapustin, P. J. Waller, J. Yang, O. M. Yaghi, *Science Advances* **2018**, *4*, eaat3198.

[8] E. Alvarez, N. Guillou, C. Martineau, B. Bueken, B. Van de Voorde, C. Le Guillouzer, P. Fabry, F. Nouar, F. Taulelle, D. de Vos, J.-S. Chang, K. H. Cho, N. Ramsahye, T. Devic, M. Daturi, G. Maurin, C. Serre, *Angewandte Chemie International Edition* **2015**, *54*, 3664-3668.
